# High-Performance Silicon Nanowire Reconfigurable Field Effect Transistors Using Flash Lamp Annealing

**DOI:** 10.1021/acsaelm.4c01896

**Published:** 2025-03-06

**Authors:** Sayantan Ghosh, Muhammad Bilal Khan, Slawomir Prucnal, René Hübner, Phanish Chava, Tom Mauersberger, Thomas Mikolajick, Artur Erbe, Yordan M. Georgiev

**Affiliations:** † Institute of Ion Beam Physics and Materials Research, Helmholtz-Zentrum Dresden-Rossendorf (HZDR), Bautzner Landstraße 400, Dresden 01328, Germany; ‡ Technische Universität Dresden, Dresden 01069, Germany; § Namlab gGmbH, Nöthnitzer Strasse 64, Dresden 01187, Germany; ∥ Technische Universität Dresden, Center for Advancing Electronics Dresden (CfAED), Dresden 01069, Germany; ⊥ Institute of Electronics at the Bulgarian Academy of Sciences, 72, Tsarigradsko chaussee blvd., Sofia 1784, Bulgaria

**Keywords:** silicon nanowire, reconfigurable FET, unipolar conduction, ambipolar conduction, flash lamp annealing, pn on-current symmetry, Schottky barrier

## Abstract

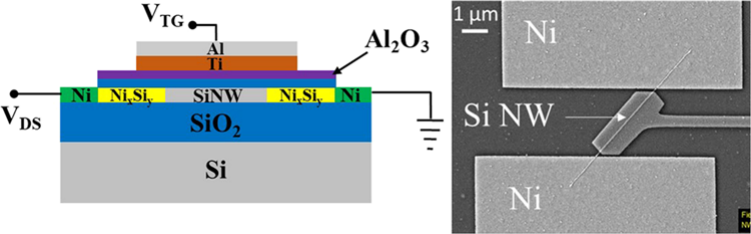

Top-down fabrication of reconfigurable field effect transistors (RFET) is a prerequisite for large-scale integration. Silicon (Si) nanowire-based RFET devices have been extensively studied in the past decade. To achieve superior RFET performance, it is necessary to develop scalable devices with controlled silicidation of the channels, a high on–off ratio, and symmetrical p- and n- on-currents. In this work, we present the electrical performance of scalable RFET devices based on Si nanowires featuring controlled silicide lengths attained through millisecond-range flash lamp annealing (FLA). The electronic properties of the transistors are optimized by tuning the different gate schemes and gate dielectric materials for nanowire passivation. We explore gate capacitive control on the energy bands in the conduction of charge carriers using various dielectric materials. The transfer characteristics of a single top-gated device with SiO_2_ as gate dielectric show enhanced ambipolar behavior with negligible hysteresis, low subthreshold swing values of 210 mV/dec, and an on–off ratio (*I*_ON_/*I*_OFF_) of up to ∼10^8^ (8 orders of magnitude). The devices also demonstrate excellent electron and hole symmetry values with a record pn on-current symmetry of 1.03. Utilizing high-performance, scalable RFET devices with elevated symmetrical on-currents holds great promise for reducing delay and power consumption in future energy-efficient integrated circuitry.

## Introduction

Transistor scaling is inevitably approaching its limit with complementary metal-oxide semiconductor (CMOS) technology entering the subnm node.^1^ Novel materials and new device concepts are being introduced to further improve device performance and downscale integrated circuitry.^2^ Therefore, advanced research and ideas are emerging based on carbon nanotubes,^3,4^ spintronics,^5^ nanowires,^6−8^ 2D electronics,^9−11^ etc. Nanowire- or nanosheet-based field effect transistors (FETs) are becoming strong contenders for ultimately scaled-down transistors. These FETs provide excellent electrostatic control over the channel with gate-all-around (GAA) or surrounding gate architectures.^12^ One such concept is known as reconfigurable FET (RFET), where the nanowire transistor can be configured to either p- or n-polarity by controlling the electrostatic potential applied at the gates.^13−17^ RFETs are based on silicide-semiconductor-silicide Schottky junctions, where the silicide supplies the charge carriers, eliminating the need for doped semiconductors. The device composition is also termed Schottky barrier FETs (SBFETs). Typical RFETs consist of an intrinsic nanowire channel that provides charge transport mechanisms for both electrons and holes. The two sides of the nanowire channel comprise the source and the drain contacts, which are metal silicides.^18^ The usual choice of metal is nickel (Ni), which forms NiSi_2_-intrinsic Si-NiSi_2_ junctions along the axis. Tunneling through this NiSi_2_-intrinsic Si Schottky barrier determines the on-current (*I*_ON_) of the RFET device controlled by either single or multiple gates. Ambipolarity is accomplished by applying an appropriate electrostatic potential to either the back gate or a single top gate, enabling n- or p-transport based on the polarity of the gate voltage. Another gate architecture consists of two independent gates placed on the two Schottky junctions. This enables a programmable unipolarity of the device. One of the gates is used to tune the device polarity, while the other gate modulates the flow of the charge carriers. One of the main challenges in RFET fabrication is to control the silicidation. Silicidation is necessary to create sharp and precisely positioned Schottky junctions, and the silicide phase and lengths must be carefully controlled during this process. Control over the silicide phase is required to form the desired silicide phases, while control over the silicide length is essential for channel scaling. This is ultimately essential for the superior performance of RFET devices. Various studies report phase control of the silicide in the nanowire channel.^19,20^ However, managing the fabrication of a precise and scalable length of the silicide intrusion has proven to be a persistent challenge. One of our previous works overcomes this by showing that the silicidation in the channel length can be controlled using a novel millisecond-range flash lamp annealing (FLA)-based silicidation technique,^21^ highlighting its potential for precise channel length definition. Building on this foundation, the present study advances the field by integrating this silicidation technique into fully functional top-down fabricated Si nanowire-based RFET devices. This work not only demonstrates the scalability of silicide channel lengths but also achieves significant improvements in device performance, including enhanced on–off current ratio, on-current symmetry, and reduced subthreshold swing. While Khan et al.^21^ primarily focused on the process development of FLA-based silicidation, this study validates its applicability for large-scale, CMOS-compatible RFET fabrication, underscoring its potential for practical applications in reconfigurable logic and technologies.

To achieve controlled silicidation, the use of a transition metal silicide is important. This is the major reason for using NiSi_2_ as the source and the drain contacts. The NiSi_2_-intrinsic Si intersection within the nanowire arrangement provides a sharp heterojunction, which can reach down to atomic levels.^13^ With such a sharp interface, better injection of charge carriers through the Schottky junction into the nanowire is possible. Also, the Fermi level of NiSi_2_ aligns near the mid-bandgap of intrinsic silicon.^22^ Due to this, the band structures can be tuned in both directions for charge carrier conduction. However, it is theoretically known that the electron Schottky barrier is slightly higher than the hole barrier (ϕ_SB,*n*_ > ϕ_SB,*p*_).^23^ This is because the NiSi_2_ Fermi level does not perfectly align itself to the mid-bandgap of intrinsic silicon.^24^ The difference in barrier height results in an asymmetry of the current levels, which presents a unique challenge. Achieving symmetry is challenging as the output voltage of one transistor may be sufficient to activate an n-FET but not enough for a p-FET due to differences in the hole mobility in silicon. This asymmetry can lead to higher operational voltage and power consumption. Furthermore, such scenarios also lead to asymmetric delay times in the circuit.^25^ The symmetrical electrical characteristics of CMOS devices are vital enablers of this technology. It is necessary to have equal threshold voltages and drive currents of n- and p-FET to fabricate energy-efficient integrated circuitry.

In traditional CMOS technology, different device dimensions are used for the p-FET and n-FET to compensate for this asymmetry. However, an RFET is a single device that can be tuned to either p- or n-polarity, making it further challenging to achieve symmetric characteristics. One approach to address this issue is modifying the silicide–Si Schottky barrier height by dopant segregation,^26−28^ which ultimately nullifies one key advantage of the RFET, i.e., the fabrication of dopant-free devices. Another approach to achieve symmetric pn characteristics is the incorporation of mechanical strain into the nanowire channel.^29^ The introduction of a dielectric layer around the nanowire is a possible solution to achieve the symmetry of electron and hole on-currents. The dielectric shell surrounding the nanowire provides a radial compressive strain, which decreases the barrier height of the electrons. This ultimately reduces the width of the tunneling barrier (the triangular shape of the barrier allows for the change in width by lowering the height), enabling the effective mass of electrons to decrease, thus increasing the electron on-current levels.^30^ The electron and hole tunneling current densities (*J*_*T*_,_*n*_, *J*_*T*_,_*p*_) through the energy barrier are assessed by assuming the Wentzel-Kramers-Brillouin (WKB) approximation,^31,32^ presented by the following equation:
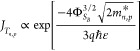
1

where *q* represents the elementary charge of the carriers, ε is the electric field created around the Schottky barrier, ℏ is the reduced Planck’s constant, and (*m*_*n*,p_^*^) is the effective mass for electrons and holes. It is inferred from the equation that by diminishing the Schottky barrier height (Φ_*SB*_) and reducing the effective masses of the charge carriers (*m*_*n*,p_^*^), the tunneling current densities for both types of charge carriers can be increased. The radial compressive strain induced by the oxide shell around the nanowire compensates for the issue of the barrier height difference and contributes to a symmetrical on-current for both electrons and holes. The additional focus of this work is the optimization and exploration of various dielectric materials like SiO_2_, Al_2_O_3_, and hBN in combination with back and multiple top gate architectures. This offers comparative insights into capacitive control of the gates over the bands for charge carrier conduction. Moreover, the electrical characteristics of the devices (employing various gating techniques) demonstrate an enhancement of the subthreshold swing (SS) values, higher p and n on-currents, and noteworthy pn on-current symmetry.

## Results and Discussion

The electrical characteristics of representative Si-based devices are shown and discussed in this section. Electrical characterizations are performed by using the back and top gate architectures. SiO_2_ and Al_2_O_3_ are used as gate dielectrics. The first measurements are performed by back gating the unpassivated nanowire-based devices. A Si carrier substrate is used as the back gate electrode. These measurements show a preliminary performance assessment of pioneering FLA-silicide unpassivated devices. Supporting Information Figure S1 shows the transfer characteristics, cross-sectional layout, and scanning electron microscopy (SEM) micrograph of one such device. The transfer characteristics are obtained by sweeping the back gate voltage (*V*_BG_) between −50 to 50 V with a step of 0.2 V in a closed loop (0 to 50 V, 50 to −50 V, and −50 to 0 V). *V*_BG_ is not increased further to avoid breakdown of the buried oxide. A drain-to-source voltage (*V*_DS_) of 0.5 V is applied for channel conduction. The device shows an ambipolar behavior with large hysteresis. The maximum currents (*I*_max_) achieved for the n- and p-type conduction are 1.4 × 10^–7^ A and 4.4 × 10^–8^ A, respectively. The threshold voltages (*V*_TH_) for the device are 2 V for p-conductance and 45 V for n-conductance. It is also noted that a high operational voltage is required to switch on the device. This is a result of using back gating, as low capacitive coupling through the 102 nm thick buried oxide ultimately degrades the subthreshold swing (SS) of the device. The SS values extracted are 3 and 1.9 V/dec for the p- and the n-branch, respectively. The large hysteresis in the transfer characteristics is attributed to dangling bonds at the nanowire surface, as well as to the high density of charged surface states in the native and buried oxide layers. These defects trap charge carriers when *V*_BG_ is applied. This charging is retained for a specific amount of time, which is called retention time.^33,34^ In addition, the existence of trapped charges at the interface of the nanowire and the buried oxide also promotes the large hysteresis and shift.^19^ Furthermore, the transfer curve shifts away from the origin (*V*_BG_ = 0 V) to a high positive *V*_BG_, which is associated with positively charged trap states at the buried oxide (BOx)–Si interface and water molecule adsorption at the surface of the nanowires.^34^ Since the measurements are performed in an ambient environment, water molecules adsorb at the surface of the nanowires. These molecules trap electrons and a positive flat band voltage builds up,^35^ resulting in a changed effective electric field. These interface-trapped charges, surface states, and hydroxyl sites can be minimized by passivating the nanowire with a dielectric shell.^36^ During the measurements, the leakage current through the oxide is also monitored to check the oxide quality. The leakage current approaches the pA range at high *V*_BG_ values.

**Figure 1:**
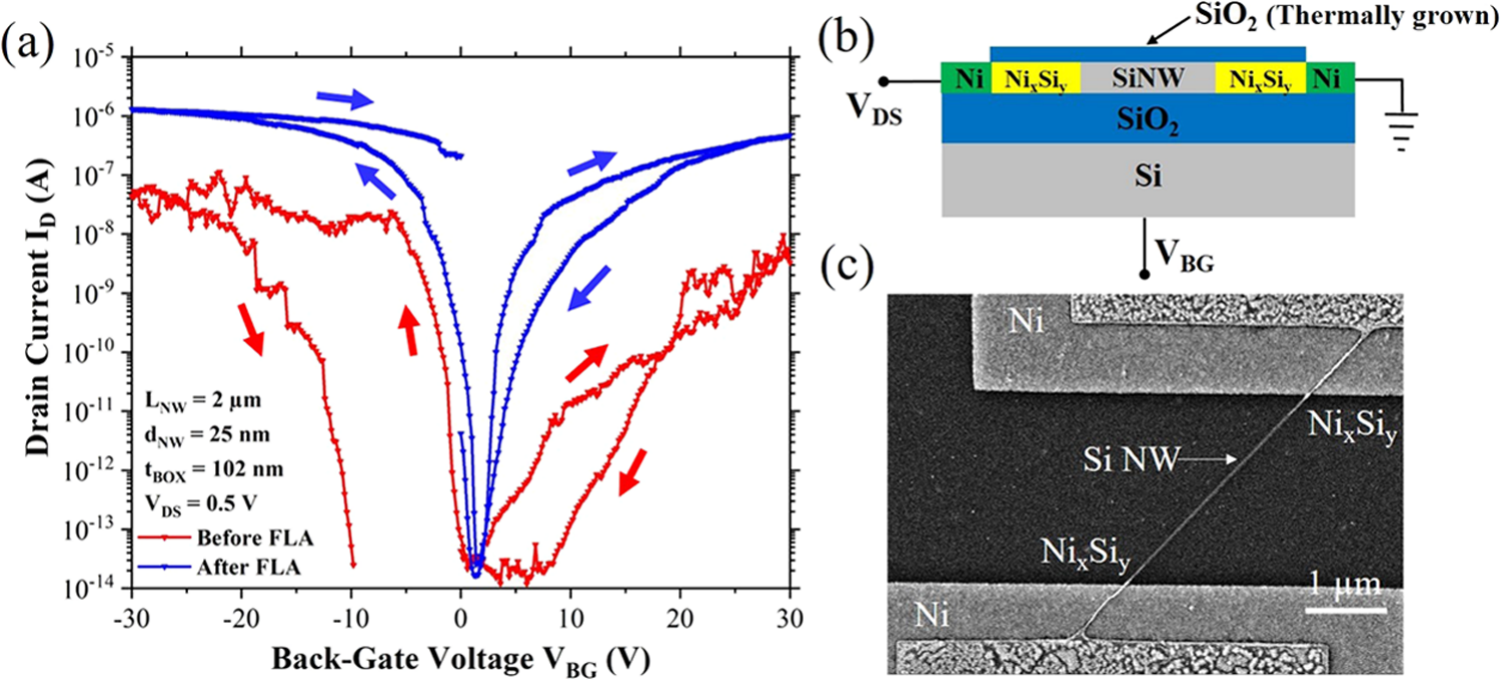
SiO_2_ passivated single-nanowire-based device: (a) back-gated transfer characteristics before and after FLA; the blue curve appears discontinued as the butterfly sweep terminates at *V*_BG_ = 0 V, reflecting the measurement protocol. The red curve’s discontinuity in the range *V*_BG_ = −10 to 0 V arises from noisy off-currents, which were excluded from the computation for clarity. (b) Cross section of the device after FLA with the voltage naming scheme. (c) SEM micrograph of the device.

The first type of passivated nanowire-based device fabricated and characterized has a 6–7 nm thermally grown SiO_2_ shell as the gate dielectric for the top gate. Thermally grown SiO_2_ is widely recognized as the most reliable and compatible dielectric for Si nanowires, owing to its superior Si-oxide interface quality, characterized by low fixed oxide charge density and minimal interface trap states.^37,38^ Oxidation of the nanowires and subsequent annealing in forming gas passivates the dangling bonds present at the surface of the nanowires.^39^ During the thermal oxidation process, oxygen (O_2_) reacts with Si to form a SiO_2_ shell, reducing their width due to Si consumption^40^ and minimizing surface and sidewall roughness induced by the dry etching process.^41^ The SiO_2_ shell around the nanowire also induces compressive strain,^42,43^ which typically impedes silicide growth.^44,45^ However, in our investigations, an increase in the silicidation length is observed compared to the silicidation lengths studied in unpassivated nanowire-based devices.^21^ This is attributed to the reduced widths and smoother surfaces of the oxidized nanowires. The silicide lengths exhibit an inverse dependence on the cross-sectional area of the nanowires, as reported in previous studies.^46−48^ SEM micrographs illustrating these findings are presented in Figure S2, where silicide intrusions of up to 350 nm are evident. Depending on specific application requirements, the silicide lengths in oxidized nanowires can be further enhanced through postoxidation annealing.^49^ In nanowire arrays, the outer nanowires exhibit longer silicide lengths compared to the inner ones, particularly in the ⟨110 ⟩ orientation array due to its smaller pitch, as shown in Figure S2d. This difference arises because the inner nanowires possess widths larger than those of the outer ones, a consequence of the proximity effect during electron beam lithography (EBL) exposure. Smaller pitch sizes amplify the proximity effect, leading to a greater variation in nanowire widths. The pitch of the ⟨100⟩ and ⟨110 ⟩ orientation arrays is 200 and 150 nm, respectively. The innermost nanowires, with a width of 55 nm, exhibit silicide lengths of ∼285 nm, while the outermost nanowires, with a width of ∼40 nm, display silicide lengths of about 345 nm. The electrical characterization of the SiO_2_-passivated devices is performed using back gating before and after the FLA step. Based on these results, devices for the fabrication of the top gates are chosen. Figure 1 depicts the back-gated transfer characteristics before and after FLA of a SiO_2_ passivated single-nanowire-based device. It also illustrates a schematic cross section and a scanning electron microscopy (SEM) micrograph of the measured device.

The transfer characteristics are obtained by sweeping *V*_BG_ between −30 to 30 V in a closed loop at *V*_DS_ of 0.5 V. The device shows improved ambipolar behavior compared to the unpassivated device. The extracted parameters from these characteristics are listed in Table 1. The shift and the hysteresis in the transfer curves are largely reduced for both measurements conducted before and after FLA. This is mainly due to the presence of the thermally grown SiO_2_ layer around the nanowire, which passivates the device by minimizing the surface states and charged hydroxyl sites.

**Table 1:** Comparison of Device Performance before and after FLA Annealing: The Si Channel in This Device Is Passivated with a Thermally Grown SiO_2_

processing step	carrier type	threshold voltage (V)	*I*_ON_/*I*_OFF_ ratio	subthreshold swing (mV/dec)	pn current symmetry
before FLA	electrons (n)	24	10^5^	2.7 × 10^2^	7.25
	holes (p)	–2.5	10^6^	360	
after FLA	electrons (n)	5	10^7^	270	2.73
	holes (p)	–2	10^8^	318	

For the results after FLA, the hysteresis is further reduced compared to before FLA results. This is mainly because FLA results in a reduced number of oxide–Si charge trapping states and the formation of an atomically sharp silicide–Si interface, significantly reducing the silicide–Si interface area which decreases charge trapping at the interface.^50^ Moreover, the annealed device shows an increase in the on-current by more than 1 order of magnitude. This enhancement is attributed to the improved electrostatic control over the atomically sharp Schottky junctions, which also results in a reduced contact resistance. The enhanced gate control over these sharp Schottky junctions facilitates finer tuning of the energy bands and band bending, leading to a more efficient charge carrier injection by tunneling. This effect contributes to a shift in the threshold voltage, effectively lowering it. Furthermore, the pn on-current symmetry is enhanced after FLA. Here, the pn on-current symmetry denotes the ratio of the maximum saturated on-current (conduction) between the p- and n-type operations of the device. It is quantified by dividing the maximum on-current of the p-conduction (usually higher due to the lower Schottky barrier height of holes compared to electrons^23^) by that of the n-conduction. A ratio value close to 1 signifies balanced performance for both p- and n-type conduction, whereas ratio values substantially greater than 1 indicate asymmetry, with one type of charge carrier (either holes or electrons) predominating the conduction. This method is employed throughout the study to calculate the pn on-current symmetry. A similar comparison of the back-gated transfer characteristics before and after FLA is conducted for SiO_2_-passivated nanowire array-based device. This is shown in the Supporting Information in Figure S3. The results reveal an increase of nearly 2.5 orders of magnitude in p-current and approximately 4 orders of magnitude in n-current after FLA. Additionally, the threshold voltages shift to lower values, and the pn on-current symmetry is significantly enhanced, approaching unity. To conclude, FLA significantly improves the device’s performance by reducing the defects present before annealing of the device.

**Figure 2:**
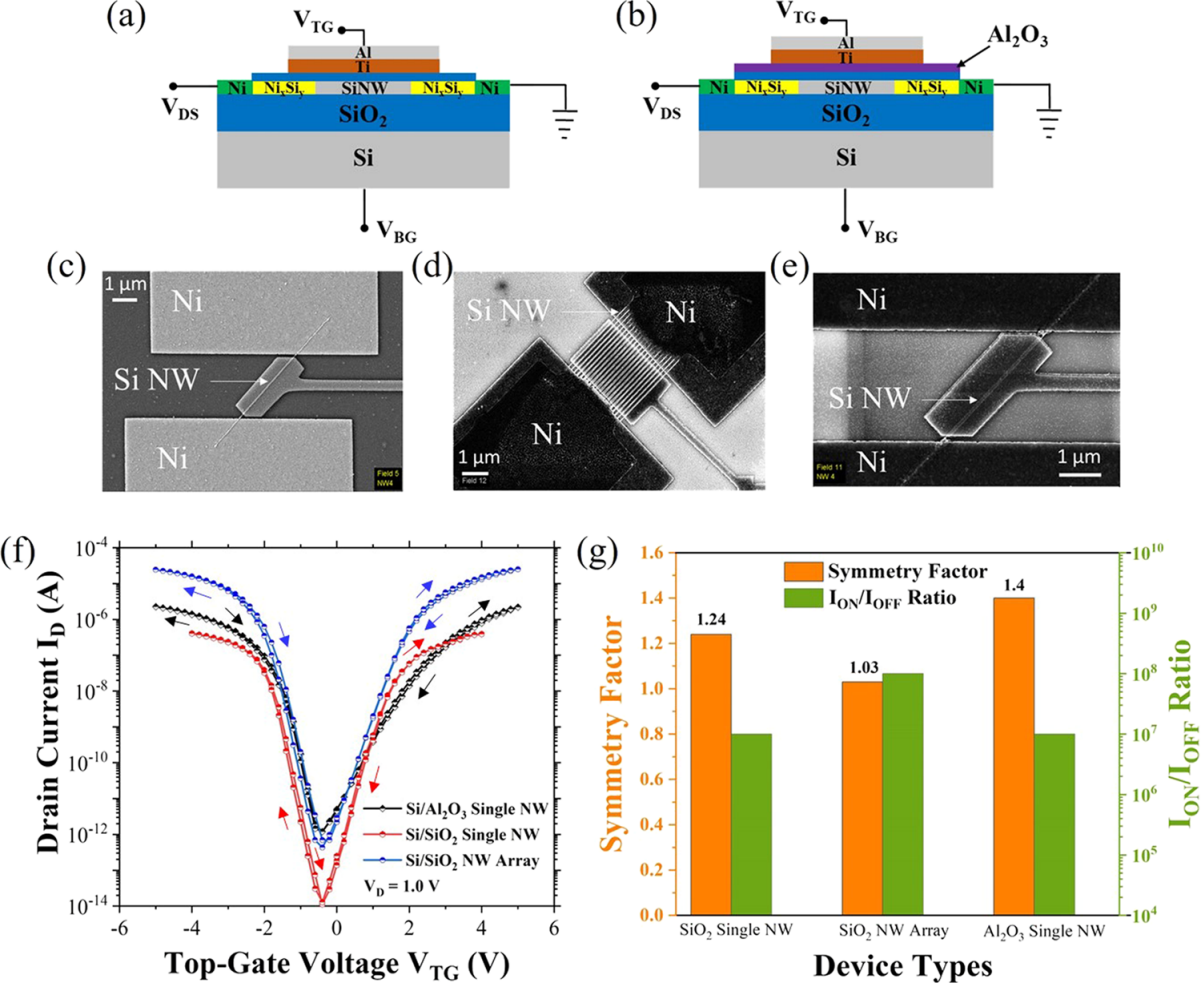
Single top-gated devices with SiO_2_ and Al_2_O_3_ passivation. Cross-sectional layout of the measurement scheme for (a) SiO_2_ passivated device; (b) SiO_2_ and Al_2_O_3_ passivated device. Top-view SEM micrograph of (c) a SiO_2_ passivated single-nanowire device with a single top gate; (d) a SiO_2_ passivated nanowire array-based device with a single top gate; and (e) a SiO_2_ and Al_2_O_3_ passivated single-nanowire device with a single top gate. (f) Comparison of SiO_2_ and Al_2_O_3_ shell-based devices showing the transfer characteristics of three different device types. (g) Histograms comparing the pn current symmetry and *I*_ON_/*I*_OFF_ ratios.

In large-scale integration of devices, a top-gate architecture is preferred, as back-gated devices require high operational voltages and cannot address individual transistor devices. Furthermore, back-gating leads to simultaneous switching on or off of all devices. Therefore, top gates are fabricated on the chosen devices. First, top-gated electrical characteristics of single-nanowire and nanowire array-based devices with SiO_2_ passivation and single-nanowire device with Al_2_O_3_ are obtained. The device schematics with voltage naming schemes, SEM micrographs, and a comparison of the electrical transfer characteristics are listed in Figure 2. For the SiO_2_ passivated single-nanowire-based device, the top gate voltage (*V*_TG_) is swept between −4 and 4 V in a closed loop. The device depicts typical Schottky barrier FET ambipolar characteristics (Figure 2f, red curve) with hysteresis lower than those shown by back-gated devices (Figure S1). The lower hysteresis is expected due to passivation of the nanowires with the SiO_2_ shell. Moreover, the device has operating voltages lower than those of back-gated devices due to a better electrostatic gate coupling through the thin thermally grown SiO_2_ shell. The better gate coupling also results in improved subthreshold slopes in these characteristics. The device shows a pn on-current symmetry value of 1.24. Furthermore, the electrical characterization of a nanowire array-based device is performed using a single top gate architecture. The transfer characteristics (Figure 2f, blue curve) are obtained by sweeping the *V*_TG_ between −5 and 5 V. For both the SiO_2_ passivated single and array-based nanowire devices, *V*_DS_ is kept constant at 1 V. Similarly, the device shows Schottky barrier FET ambipolar type behavior. The hysteresis is marginally larger than that of a single-nanowire device. This is expected as the array consists of 20 nanowires in parallel, with each nanowire having the SiO_2_ dielectric shell. The defects in the oxide and at the oxide–Si interface of each nanowire add up, which results in a larger hysteresis. Moreover, the increase in the *I*_ON_ is expected because there are 20 nanowire channels in these devices. Additionally, the channel lengths in the array are 2.3 μm, while the channel length in the single-nanowire device is 2.5 μm. These shorter lengths lead to a further increase in the *I*_ON_. An excellent pn current symmetry value of 1.03 is shown by this device, which is the best result achieved for scalable reconfigurable FETs with FLA. The effective Schottky barriers for the electrons and holes (Φ_*SB*,*n*_ and Φ_*SB*,*p*_) are also extracted by the activation energy method (further described in Supporting Information Figure S4). Values of Φ_*SB*,*n*_ and Φ_*SB*,*p*_ are 200 and 160 meV, respectively. These values are extracted from the transfer curves obtained at *V*_DS_ = 1 V. The lower value of Φ_*SB*,*p*_ reinforces the better subthreshold swing shown by the p-branch. The calculated values for interface charge density (*Q*_it_) and trap density (*D*_it_), representing the approximate concentration of charges and trap states at the SiO_2_-passivated nanowire interface, are *Q*_it_ ∼ 1.6–1.9 × 10^–6^ C cm^–2^ and *D*_it_ ∼ 1–1.2 × 10^13^ cm^–2^ eV^–1^ for n-type conduction and *Q*_it_ ∼ 1.2–1.4 × 10^–6^ C cm^–2^ and *D*_it_ ∼ 8–9 × 10^12^ cm^–2^ eV^–1^ for p-type conduction.

The next type of device consists of a single silicon nanowire with a dielectric stack of ca. 1–2 nm thermally grown SiO_2_ (as a passivation layer) and ca. 6–7 nm Al_2_O_3_ deposited by atomic layer deposition (ALD). To enhance the RFET performance, it is important to have a better field effect capacitive coupling over the Schottky junctions and the nanowire channel. This can be achieved by lowering the oxide thickness, but this increases the risk of a higher gate leakage current. However, with high-k dielectric materials, such as Al_2_O_3_, such issues can be tackled. The capacitance of structures containing high-k materials is larger than that of SiO_2_ at the same oxide thickness. In turn, thicker insulator layers can be used when SiO_2_ is replaced with a high-k material. The main factor for using Al_2_O_3_ over SiO_2_ is that it has a higher dielectric constant (*κ* ∼ 9), good thermal stability, and a large equivalent bandgap.^51^ It also provides a large bandgap offset for the conduction and the valence band similar to that of SiO_2_.^52^ However, in terms of surface passivation, the silicon nanowire with Al_2_O_3_ as a gate dielectric has a higher density of surface states compared to only thermally grown SiO_2_.^53^ To remedy this, an interfacial thin passivating layer of SiO_2_ is grown. An alternative dielectric material suitable for this work is HfO_2_, which offers advantages such as a higher dielectric constant (enhancing electrostatic gate coupling) and excellent thermal stability, potentially improving device performance.^54,55^ However, compared to HfO_2_, Al_2_O_3_ provides a higher bandgap energy, resulting in superior leakage current suppression and better surface passivation due to reduced defect density.^56^ A trade-off is necessary, and Al_2_O_3_ is chosen due to its availability. Nonetheless, Si nanowire-based RFETs with HfO_2_ are also a promising option for exploration. The silicidation of nanowires with an Al_2_O_3_ shell shows significant variability with inconsistent silicide lengths across devices. The best results, shown in Figure S6, feature the most symmetric and longest silicide intrusions but are still shorter than those observed with SiO_2_ shells, likely due to the higher stress induced by the Al_2_O_3_ shell. SEM analysis reveals a rough nanowire surface after Al_2_O_3_ deposition and silicidation, emphasizing the need for further process optimization.

The transfer characteristics for the Al_2_O_3_-based device are obtained using either a back gate (shown in Supporting Information Figure S7) or a single top gate. Figure 2b shows the cross-sectional layout, Figure 2e shows the SEM micrograph, and Figure 2f (black curve) shows the electrical characteristics of a single-nanowire-based device with SiO_2_–Al_2_O_3_ shell, with an effective oxide thickness (EOT) of ca. 5 nm. The transfer characteristics are obtained by sweeping the voltages between −5 and 5 V in a closed loop at a constant *V*_DS_ of 1 V. The device depicts improved ambipolar characteristics similar to the SiO_2_ passivated single top-gated device (Figure 2f, red curve). However, since the overall physical thickness of the stacked dielectric layer (SiO_2_ and Al_2_O_3_) is larger than the thickness of only SiO_2_, a higher voltage sweep is possible without risking dielectric breakdown. Similar to the previous devices, it is evident from the plot that the whole transfer curve shifts to a low negative voltage (with *I*_OFF_ minima around *V*_TG_ = −1 V). It is observed that the hysteresis for both branches is almost negligible, inferring a low density of surface states at the interface of the nanowire and the passivating SiO_2_ layer. The approximate calculated values of *Q*_it_ and *D*_it_ for a single-nanowire-based device with a SiO_2_/Al_2_O_3_ shell are *Q*_it_ ∼ 4 × 10^–6^ C cm^–2^ and *D*_it_ ∼ 3 × 10^13^ cm^–2^ eV^–1^ for n-type conduction and *Q*_it_ ∼ 1.7 × 10^–6^ C cm^–2^ and *D*_it_ ∼ 1 × 10^13^ cm^–2^ eV^–1^ for p-type conduction. A comparison of the extracted electrical parameters of single-nanowire and nanowire array-based devices with SiO_2_ passivation and single-nanowire device with Al_2_O_3_ is presented in Table 2.

**Table 2:** Extracted Parameters from the Ambipolar Transfer Characteristics of Single-Nanowire and Nanowire Array-Based Devices with SiO_2_ Passivation and a Single-Nanowire Device with Al_2_O_3_ Passivation at *V*_DS_ = 1 V

nanowire devices	carrier type	threshold voltage (V)	*I*_ON_ (A)	*I*_ON_/*I*_OFF_ ratio	subthreshold swing (mV/dec)	mobility (μ) (cm^2^/(V s))	pn current symmetry
Si-SiO_2_ single nanowire	electrons (n)	0.75	3.3 × 10^–7^	∼10^7^	260	29.6	1.24
	holes (p)	–1.5	4.1 × 10^–7^	∼10^7^	210	28.3	
Si-SiO_2_ nanowire array	electrons (n)	2	2.3 × 10^–5^	∼10^8^	306	77	1.03
	holes (p)	–2	2.4 × 10^–5^	∼10^8^	228	69	
Si-SiO_2_–Al_2_O_3_ single nanowire	electrons (n)	2.3	1.3 × 10^–6^	∼10^7^	469	242	1.4
	holes (p)	–3.5	1.94 × 10^–6^	∼10^7^	200	68	

It should also be noted that the off-currents (*I*_OFF_) of the devices fall within the picoampere (pA) to femtoampere (fA) range, classifying them as low off-currents. The nanowire array-based device exhibits a slightly higher *I*_OFF_ compared with the single-nanowire-based device. This increase is attributed to the cumulative effect of 20 nanowires in the channel, resulting in a higher probability of charge carrier conduction under the influence of the negligible gate voltage and applied *V*_DS_. Also, the on-current levels for the Al_2_O_3_ passivated single-nanowire device are recorded to be higher than those of the SiO_2_ passivated single-nanowire device (Figure 2f). Both of these devices have the same crystallographic orientation of the nanowires. This is reasonable, knowing that the device with the high-k dielectric shell provides better gate coupling and tuning of barriers. However, the moderately higher off current of the device compensates for this, resulting in a similar *I*_ON_/*I*_OFF_ ratio with respect to the SiO_2_-passivated device (Figure 2g). This comparable *I*_ON_/*I*_OFF_ ratio of SiO_2_ and Al_2_O_3_ passivated devices with different dielectric thicknesses is plausible since the estimated EOT for the Al_2_O_3_ device is 5 nm which is almost the same as for the SiO_2_ device (∼6 nm). Furthermore, the Al_2_O_3_-based device shows a marginally larger shift of the minimum away from *V*_TG_ = 0 V with a higher off current. Comparing the subthreshold swing of the devices, the lowest value is achieved for the p-branch along with the highest for the n-branch in the Al_2_O_3_-based device. With high-k dielectric material as the gate oxide, the device is expected to have a higher capacitive top gate coupling. This should ultimately improve the subthreshold swing for both types of conduction. Though the p-type swing shows the best value, the n-branch is increased compared to that of the SiO_2_ passivated devices. This could be due to the dual dielectric oxide shell around the nanowire providing two interfaces (the Si-SiO_2_ and the SiO_2_–Al_2_O_3_). This leads to a higher possibility of defects, depending on the quality of the films deposited. Such defects can eventually degrade the top gate field effect, deteriorating the subthreshold swing. To further investigate this, the effective Schottky barriers for electrons and holes (Φ_*SB*,*n*_ and Φ_*SB*,*p*_) are extracted by the active energy measurements method (also shown in Supporting Information Figure S5). Values of Φ_*SB*,*n*_ and Φ_*SB*,*p*_ are 321 and 247 meV, respectively. The p-branch has a better subthreshold swing due to the lower value of Φ_*SB*,*p*_. Furthermore, the SiO_2_–Al_2_O_3_ shell appears rougher in the SEM investigation. The optimization of the Al_2_O_3_ deposition and the subsequent silicidation process must be undertaken for superior device performance. It is also observed that the pn symmetry factor for Al_2_O_3_ passivated single-nanowire-based devices is slightly higher compared to that of the SiO_2_ passivated single-nanowire-based device. This is attributed to the differences in compressive strain induced by the SiO_2_ layers in both the devices. The SiO_2_ layer grown around the nanowire generates compressive strain, which effectively reduces the tunneling masses, particularly for electrons, thereby enhancing n-type currents and promoting symmetry.^29^ The extent of compressive strain depends on the thickness of the SiO_2_ layer: thinner layers induce a lower intrinsic compressive strain, while thicker layers produce a higher strain. Symmetry in the device performance is governed by the ability to balance the modulation of p-type and n-type currents. In the case of Si-SiO_2_-based single-nanowire devices, a SiO_2_ layer with a thickness of ca. 6–7 nm is grown, providing sufficient strain to achieve better symmetry. In contrast, for the Si-SiO_2_–Al_2_O_3_-based single-nanowire device, SiO_2_ is primarily used as a passivating layer, and only a thin layer (ca. 1–2 nm) is grown before ALD deposits the Al_2_O_3_ layer. This reduced SiO_2_ thickness results in lower compressive strain, which diminishes the modulation of the effective electron tunneling mass, ultimately leading to a degraded symmetry factor compared to the Si-SiO_2_-based device. The nanowire array-based device with a SiO_2_ shell shows an excellent pn current symmetry of 1.03 with a high *I*_ON_/*I*_OFF_ ratio of 10^8^.

**Figure 3:**
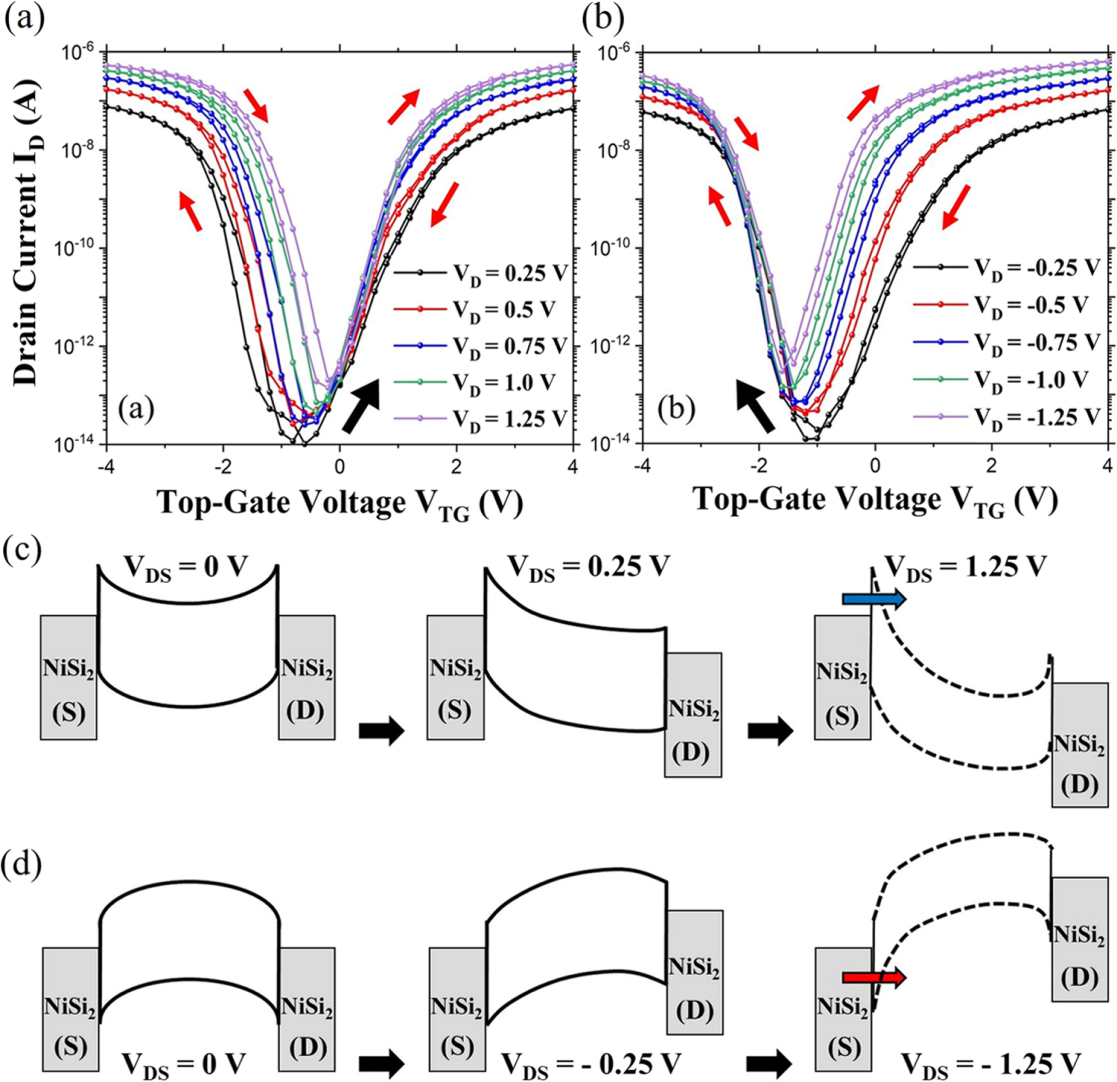
Transfer characteristics shift analysis of the single-nanowire-based device with a SiO_2_ shell. The different shifts in the transfer curve are seen with (a) positive and (b) negative values of *V*_DS_. The length and width of the nanowire are 2.5 μm and 25 nm, respectively. (c) and (d) Band diagrams illustrating the drain-sideband bending in the single-nanowire-based device for incrementing *V*_DS_ values. (c) At *V*_TG_ > 0 V and *V*_DS_ = 0.25 V, band bending occurs predominantly at the drain side, facilitating electron conduction. As the positive *V*_DS_ increases, the field coupling intensifies, narrowing the energy barrier at the drain side and shifting the off-state current for the n-branch. (d) At *V*_TG_ < 0 V and *V*_DS_ = −0.25 V, the electric field coupling at the drain side increases due to the negative *V*_DS_, causing a reduction in the barrier width. This promotes hole conduction, resulting in a shift of the off-state current for the p-branch. The band bending becomes more pronounced as *V*_DS_ is incremented negatively, directly influencing the hole injection. Additionally, *V*_DS_ also influences the entire band under the effect of *V*_TG_, inducing band bending on the source side, which enhances the tunneling current and ultimately supports charge carrier conduction.

The top-gated transfer characteristics (*I*_D_-*V*_TG_) for varying *V*_DS_ and the output characteristics (*I*_D_-*V*_DS_) for varying *V*_TG_ for both the single and the nanowire array-based device with SiO_2_ passivation and single-nanowire-based device with Al_2_O_3_ are shown in Supporting Information Figure S8. For the output characteristics, a distinct Schottky-type behavior is evident for the device configuration at elevated *V*_DS_ with an increase in *I*_D_ values for increased *V*_TG_. This provides a comprehensive assessment of the contact properties grounded in the ambipolar shape observed in the transfer characteristics. With higher *V*_TG_, there is a stronger band bending at the source and the drain Schottky junctions, providing higher tunneling of charge carriers. This increases the *I*_D_ values due to a higher current flow. For the transfer characteristics with varying *V*_DS_, for all of the device types, the transfer curves shift to a negative voltage (minima centered around *V*_TG_ ∼ −1 V). To understand the shift, the characteristics are obtained at different values of *V*_DS_, where *V*_DS_ increases from −1.25 to 1.25 V in steps of 0.25 V. The results are presented in Figure 3.

For simplicity of explanation, transfer curves for negative and positive *V*_DS_ are presented separately. In the case of the top-gated devices, the positioning of these curves is mainly dependent on the device fabrication process. The e-beam evaporation of the metals generates secondary electrons and characteristic X-rays. This results in the induction of charges in the dielectric layer and buried oxide, which leads to carrier trapping.^57^ In Figure 3a (b), it is seen that the minimum of the transfer curve shifts toward (away from) the origin by increasing (decreasing) the *V*_DS_ value, respectively. This type of characteristic is common for such Schottky FETs.^58^ This phenomenon leads to the fact that an increase of *V*_DS_ to the positive values (*V*_DS_ > 0), moves the minimum of the curve to a higher *V*_TG_, while a decrease in *V*_DS_ to negative values (*V*_DS_ < 0), shifts the minimum to a lower *V*_TG_. The reason for this can be that the minimum off current (*I*_OFF_) depends on *V*_DS_ and that its value rises with an increase in *V*_DS_. As *V*_DS_ increases in either direction, there is band bending at the drain side for electron conduction and at the source side for hole conduction, respectively. When *V*_DS_ is raised in a positive direction (Figure 3a), the electric field coupling increases at the drain side, reducing the barrier width. This causes *I*_OFF_ to rise, shifting the n-branch. However, since increasing *V*_DS_ positively does not influence the source side, the p-branch remains unaffected.^35^ The opposite scenario occurs during hole conduction when *V*_DS_ is decreased, as shown in Figure 3b. The *I*_OFF_ minimum is also dependent on the work function of the gate metal and can be shifted in either direction depending on the metal used. The shift in the minimum also relies on the bandgap energy of the channel material. Hence, by using lower bandgap semiconductors like germanium, this shift can be controlled.^58^

**Figure 4:**
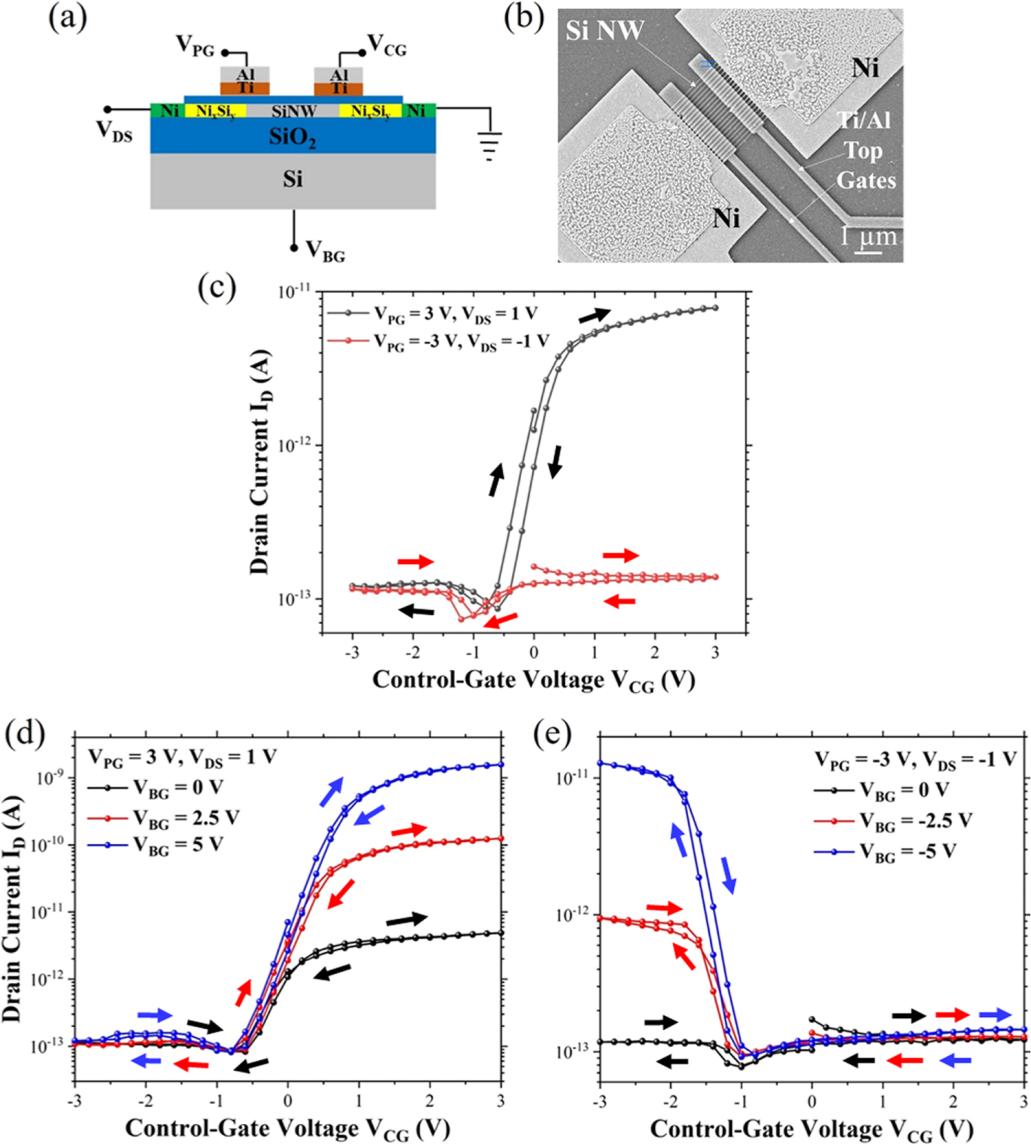
Two-top-gated nanowire array-based device with a SiO_2_ shell. (a) Cross-sectional schematic layout of the two-top-gated device with corresponding voltage naming schemes. (b) SEM micrograph of the measured device. (c) Transfer characteristics for unipolar operation. Unipolar device characteristics modulation with auxiliary back gating leads to enhanced transfer characteristics of the device showing (d) n-type conduction and (e) p-type conduction under the influence of supplementary back gating.

Two or more top gates are required to tune the unipolarity of the devices. One of the gates, the program gate (PG), programs the device polarity, and the other gate, the control gate (CG), modulates the flow of the charge carriers. The transfer characteristics, schematic layout, and SEM micrograph of two top-gated devices are presented in Figure 4. The device is tuned to p-polarity by keeping *V*_DS_ at −1 V and program gate voltage (*V*_PG_) at −3 V. The control gate voltage (*V*_CG_) is swept between −3 and 3 V in a closed loop to modulate the flow of the holes. The device shows only marginal modulation of the hole current. The device is then tuned to n-polarity by keeping *V*_DS_ at 1 V and *V*_PG_ at 3 V. *V*_CG_ is again swept between −3 and 3 V. Here, the device shows a higher modulation of the electron current in comparison to the hole current. It is seen that the transport regime with dominant thermionic emission is present in the range from −1 to 2 V, while the tunneling dominant on-state is observed beyond 2 V.

Moreover, it is seen that the transfer curves center away from *V*_CG_ = 0 V. This phenomenon is similar to the one observed for single-top-gate characteristics. As stated, one of the reasons for this mismatch between electron and hole modulation can be the choice of the gate metal stack. Titanium nitride (TiN) can circumvent this issue as its work function can be tuned to avoid the shift.^59^ For both types of charge carrier conduction, the generated on-current levels are low with respect to those of the single-top-gated device. This is particularly evident in the minimal modulation of the p-type current as a function of the gate voltage. There are some possible explanations for this scenario. First, the induction of the charge carrier traps and interface states in the ungated area of the nanowire leads to the formation of an energy barrier in the middle of the channel. This effect is shown for intrinsic Si nanowires by Cui et al.,^60^ whereby the phenomenon could be due to a weak top gate control in the ungated regions. The negative nature of these induced charges in the ungated regions provides a stronger adverse effect on hole conduction and modulation. Another reason for the low on-currents can be a slight misalignment of the individual top gate structures, ultimately missing the region where the Si and the NiSi_2_ region are in contact. Such misalignment can lead to weaker gate coupling over the Schottky junction, limiting the current conduction. This can be solved by fabricating wider top gates and the proper passivation of the nanowire channel. Another way the issues can be addressed is by using the back gate as a supplementary program gate.

To further investigate the weak modulation, auxiliary back gating is used in addition to the top gates. This enhanced modulation helps to improve the electrostatic control of the channel and to investigate the previously observed very low p-branch obtained by using the two-top-gate architectures. The transfer characteristics and measurement schematic are presented in Figure 4d,e. An increasing modulation of the charge carriers is seen with increasing back gate voltages. The p- and n-branch depict improved *I*_ON_/*I*_OFF_ ratios of up to 10^2^ and 10^4^, respectively. The current levels are still low in comparison to the single top-gated devices. This can be due to the weak electrostatic control by the two top gates leading to fewer charge carrier injections through the silicide–Si Schottky junctions.

**Figure 5:**
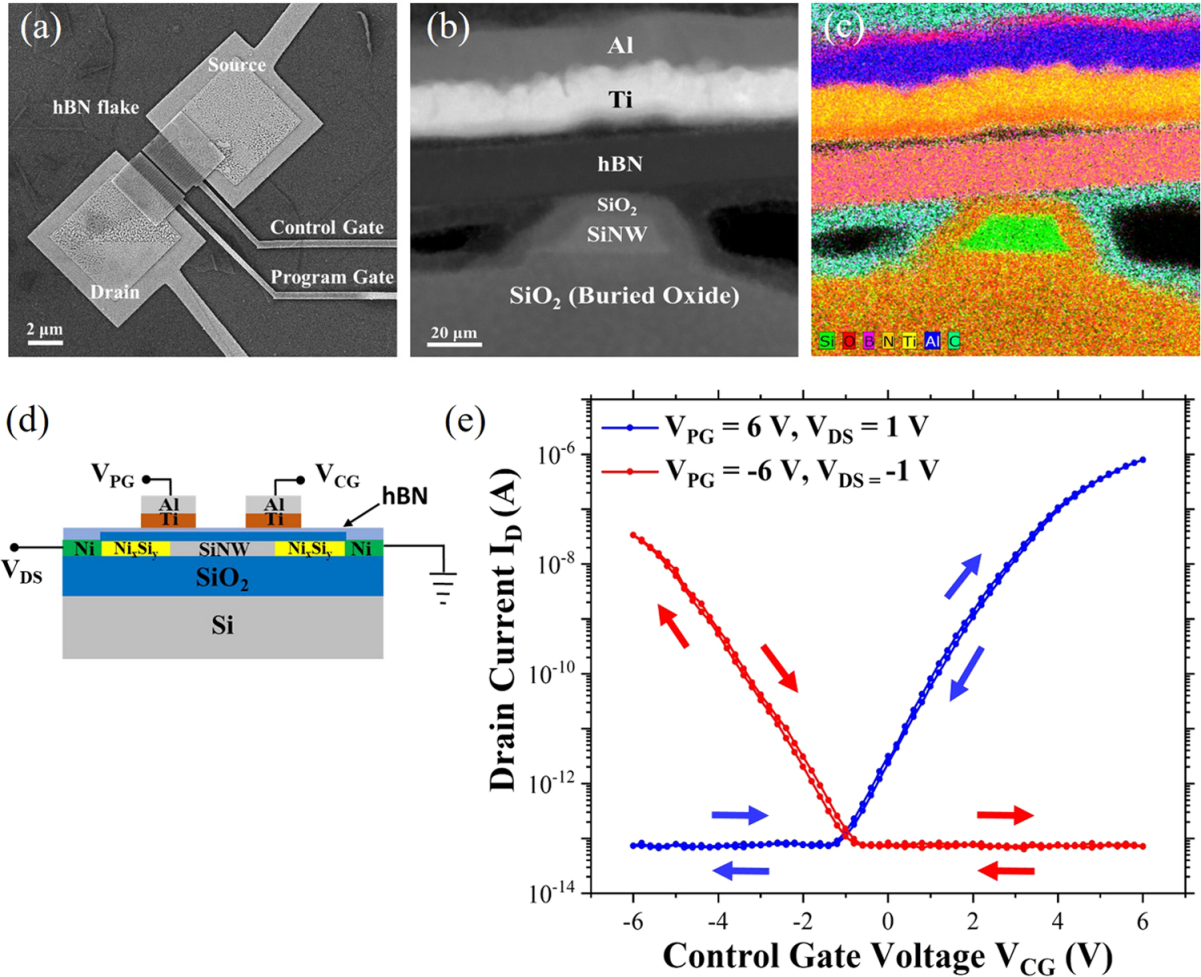
Improved unipolar two-top-gated device characteristics of a nanowire array-based device. (a) Top-view SEM micrograph of the two-top-gated nanowire array device with the source-drain contact pads, hBN flake transferred on top, and the broader CG and PG structures; (b) Cross-sectional HAADF-STEM micrograph of the sectioned nanowire. (c) Corresponding superimposed EDXS-based element distribution map of the device. The device structure shows the presence of a buried oxide layer under the silicon nanowire. 6–7 nm of SiO_2_ serves as the primary dielectric layer, while hBN is present as an additional passivating and encapsulating layer. Ti and Al metal stacks serve as the gate electrode on top of the nanowire. (d) Cross-sectional schematic of the device including the voltage naming conventions used in this work. (e) Two-top-gated unipolar transfer characteristics of the device consisting of a nanowire array of 20 nanowires with a SiO_2_ shell. The length and width of the nanowires are 2.3 μm and 25 nm, respectively.

To improve the unipolar characteristics, a new RFET device is fabricated, allowing for better passivation of the nanowires by minimizing the charge carrier traps and interface states. To this end, the 2D dielectric material hexagonal boron nitride (hBN) is used as an additional dielectric layer above the SiO_2_ shell. Our previous work illustrates the excellent passivation and dielectric properties of hBN in the context of silicon nanowires.^16^ The utilization of hBN as a passivating layer proved effective in addressing the issues previously raised. First, it prevents the induction of charges (secondary electrons and X-rays generated during e-beam evaporation of the top gate metals) in the ungated regions of the nanowire. This ultimately results in a reduced energy barrier in the center of the nanowire channel. Second, the transfer of an exfoliated thin hBN flake (∼20 nm) over the device not only encapsulates it from the environment but also helps to fabricate wider top gates on the device. This reduces the chance of misalignment of the gate electrode with respect to the Schottky junctions. Furthermore, broader top gates offer enhanced capacitive coupling (due to a larger gate electrode area) across the junction areas, which is required for further band bending and better modulation of the charge carriers. The device is characterized by top-down SEM imaging (Figure 5a) and cross-sectional transmission electron microscopy (TEM)-based analysis (Figure 5b,c). The TEM inspection shows the hierarchy of the sectioned device. The sectioning of the device is carried out perpendicular to the Si nanowire axis. As seen in Figure 5b, the nanowire channel (sitting above the buried oxide layer) displays a trapezoidal shape with a height of 20 nm and a width of 25 nm. The high-angle annular dark-field (HAADF) scanning TEM (STEM) image also confirms the ca. 6–7 nm thick thermally grown SiO_2_ shell around the nanowire structure. The thickness of the hBN layer is approximately 20 nm, encapsulating the nanowire from the top. Above the dual dielectric layer, a combination of titanium (Ti) and aluminum (Al) forms one of the gate electrodes of the device. These observations are further confirmed by studying the element distributions in the sectioned device by using spectrum imaging analysis based on energy-dispersive X-ray spectroscopy (EDXS), as shown in Figure 5c. The presence of oxide around the nanowire results from controlled thermal oxidation, while the oxide traces between the metal gates arise from breaking the vacuum during deposition, which likely occurs under ambient conditions. The EDXS map also confirms black areas, which are presumably empty spaces (voids). Carbon (C) is detected atop the Ti–Al gate stacks resulting from the protective capping layer applied during TEM specimen preparation. However, the C layer present near the cross section of the nanowire channel, above the SiO_2_ layer, and beneath the hBN dielectric layer stems from the device fabrication process. To image the device during its fabrication process, several SEM scans are undertaken, causing organic substances present on the sample’s surface to fracture and subsequently deposit as carbon layers.^16^

For the electrical characteristics, Figure 5d shows the cross-sectional schematic layout of the improved device and Figure 5e shows the two-top-gated transfer characteristics. A distinct and well-improved unipolar characteristic is visible for both n- and p-conduction. For modulating the n-branch, *V*_CG_ is swept by a butterfly sweep from −6 to 6 V while keeping the *V*_PG_ constant at 6 V (for blocking the hole conduction) and *V*_DS_ constant at 1 V. The opposite polarity is maintained for the p-conduction. It is evident from the plot that both the transfer curves shift to a low negative voltage with a minimum of around −1 V. However, compared to the previous two top-gated measurements, the characteristics show much higher on-current levels and almost negligible hysteresis. The use of slightly higher *V*_CG_ (compared with the previous measurements) is due to the use of two dielectric layers. Therefore, a slightly higher voltage sweep is required for the modulation of the charge carriers. This also has a relatively low capacitive coupling effect on the channel (compared to the single top gate devices), increasing the values of the subthreshold swing of the devices. The extracted parameters for the unipolar two top-gated device are depicted in Table 3. The methods and equations used to calculate the various device performance parameters are detailed in the Supporting Information.

**Table 3:** Extracted Parameters from the Unipolar Transfer Characteristics of the Nanowire Array-Based Device at *V*_DS_ = 1 V^a^

branch type	*I*_ON_ (A)	threshold voltage (V)	*I*_ON_/*I*_OFF_ ratio	subthreshold swing (mV/dec)	mobility (cm^2^/V·s)	pn current symmetry
n-branch	7.9 × 10^–7^	4	10^7^	750	329	∼2.3
p-branch	3.3 × 10^–8^	–5	10^5^	850	20	

a The device has two top gate electrodes: a program gate and a control gate. SiO_2_ is used as the primary gate dielectric, and hBN is used as a passivating and encapsulating dielectric layer.

In this work, the performance of the presented top-down fabricated Si nanowire RFET using FLA is compared with those of the best state-of-the-art Si nanowire RFET devices from the literature. The key device parameters, including *I*_ON_-*I*_OFF_ current ratio, maximum on-current, subthreshold slope, and pn current symmetry, are summarized and compared in Table 4, highlighting the advancements achieved in this study. The optimal device parameters achieved in this work demonstrate exceptional performance across several key metrics. Notably, the *I*_ON_-*I*_OFF_ current ratio of 10^8^, achieved equally in both n-type and p-type operations, outperforms the devices reported by Heinzig et al. (2012) and Simon et al. (2022). Heinzig et al. (2012) pioneered RFETs using bottom-up grown nanowires, while this work utilizes a CMOS-compatible top-down fabrication approach with SOI substrates. This method offers better control, scalability, and integration with existing semiconductor technologies. The maximum on-currents of 2.3 × 10^–5^ (n-type) and 2.4 × 10^–5^ A (p-type) are also significantly higher than those of earlier devices, such as those reported by Simon et al. (2017) and Jeon et al. (2021). The subthreshold slopes (SS) of 260 mV/dec (n-type) and 210 mV/dec (p-type) are balanced and comparable to those of Simon et al. (2017), reporting values of 134 mV/dec (p-type) and 245 mV/dec (n-type). The improved current symmetry of 1.03 also highlights the best performance of the presented device, which benefits from the utilization of a high-quality dielectric stressor layer and FLA for silicidation, offering more uniform and controllable silicide formation compared to conventional rapid thermal processing (RTP) and rapid thermal annealing (RTA).

**Table 4:** Comparison of Extracted Parameters for Various State-of-the-Art RFET Devices from the Literature^a^

**parameter**	Heinzig et al.^13^ (2012)	Simon et al.^58^ (2017)	Simon et al.^61^ (2022)	Jeon et al.^62^ (2021)	this work (2024)
on/off	1 × 10^9^ (p)’	symmetric,	890 (p)	∼10^6^ (p)	10^8^ (p)
current ratio	6 × 10^7^ (n)	not reported	350 (n)	∼10^6^ (n)	10^8^ (n)
maximum	1.9 μ A (p)	5.33 μ A (p)	18 μ A/μ m (p)	∼1 × 10^–7^ A (p)	2.4 × 10^–5^ A (p)
on-current	1.1 × 10^–7^ A (n)	26.6 μ A (n)	10 μ A/μ m (n)	∼2 × 10^–8^ A (n)	2.3 × 10^–5^ A (n)
subthreshold	90 (p)	134 (p)	300 (p)	not reported	210 (p)
slope (mV/dec)	220 (n)	245 (n)	308 (n)		260 (n)
fabrication	bottom-up	top-down	top-down	bottom-up	top-down
process	VLS grown	(SOI)	(SOI, EBL)	CVD grown	(SOI, EBL)
annealing	RTP	RTP process	RTA	RTP process	FLA process
method	process	450 °C for 15 s	process	500 °C for 30 s	3.6 kV, 6 ms
pn current symmetry	17.27	1.18	1.8	5	1.03

a The devices differ in the fabrication process, annealing methods, and overall device performance metrics, including the *I*_ON_-*I*_OFF_ current ratio, maximum on-current, subthreshold slope (SS), and pn on-current symmetry.

**Figure 6:**
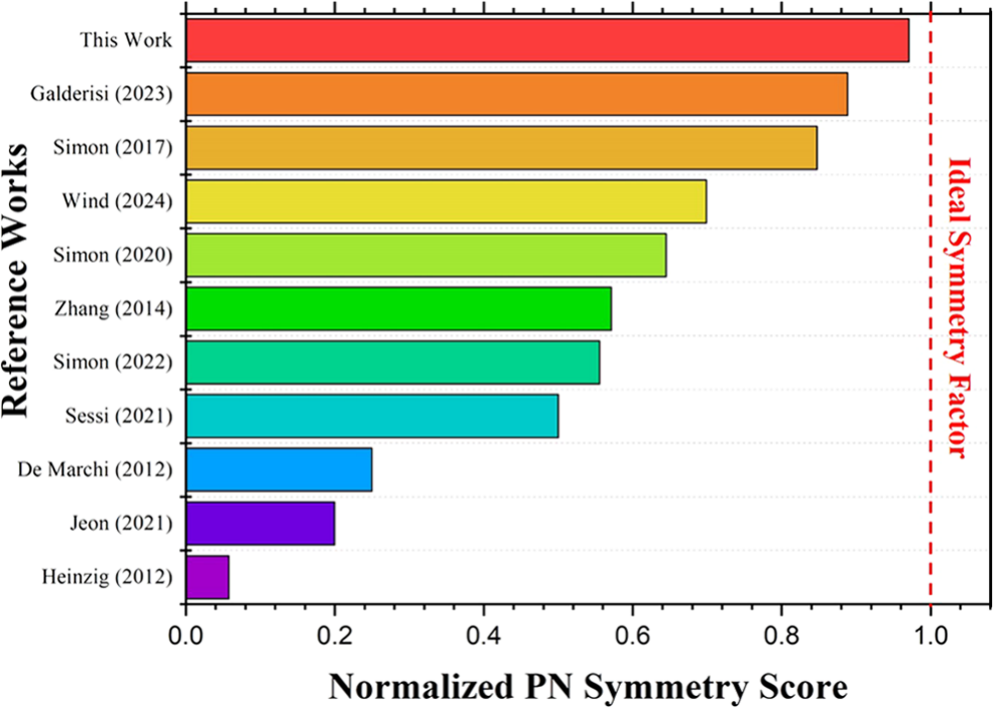
Normalized pn symmetry scores for various RFET studies over the past decade are presented,^13,18,58,61−67^ where a higher score indicates closer proximity to the ideal pn symmetry value of 1. The device from this work is highlighted in red, demonstrating its superior symmetry performance compared to other benchmark RFET devices.

The best device performance, including pn on-current symmetry, *I*_ON_/*I*_OFF_ ratios, and pn on-currents presented in this study, is compared to other benchmark RFET devices fabricated over the past decade. In Figure 6, the pn symmetry factors are compared after normalization to highlight the best result, which is closest to the ideal factor of 1. The best-performing device in this work is marked in red to emphasize its strong performance in achieving near-ideal symmetry. The normalized symmetry score is calculated using the following equation:
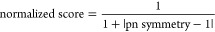
2

where pn symmetry represents the measured pn symmetry value from the experiment or literature. |pn symmetry −1| denotes the absolute deviation from the ideal symmetry of 1. The fraction ensures that values closer to 1 receive higher scores, making them more favorable in comparison.

**Figure 7:**
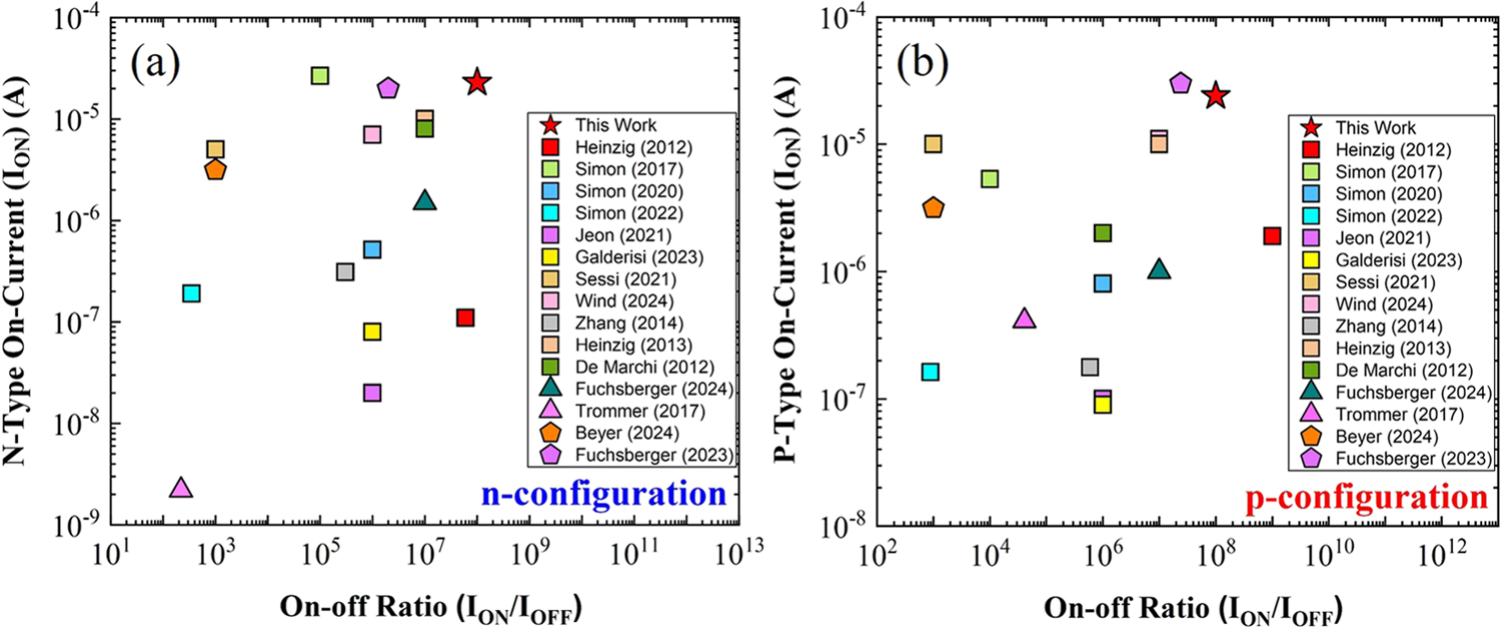
Benchmark comparison of RFET devices in terms of on-current versus on–off ratio for (a) n-type and (b) p-type configurations. The performance of the device fabricated in this work is compared to other RFET devices developed over the past decade, including foundational studies. The star symbol represents the best device result from this work, while square symbols indicate RFETs based on Si nanowire channels,^13,30,58,61−67^ triangles denote Ge-based RFETs,^68,69^ and pentagons represent SiGe-based RFETs.^70,71^.

Furthermore, Figure 7 illustrates the relationship between the on-current and the *I*_ON_/*I*_OFF_ ratio for both n-type and p-type configurations, enabling a comparative analysis of various devices, including state-of-the-art RFETs based on Si, germanium (Ge), and silicon–germanium (SiGe). The device fabricated in this work demonstrates a well-balanced performance between the on-current and the on–off ratio, exhibiting significantly high values for both types of charge carriers.

Based on the calculated device performances, an estimation for reducing delay times and power consumption in RFET-based circuits is derived. The expression for the delay time (*t*_delay_) and the dynamic power consumption (*P*_dynamic_) can be written as^72,73^
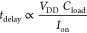
3
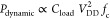
4where *V*_DD_ is the supply voltage, *C*_load_ is the load capacitance, *I*_on_ is the on-current, and *f*_c_ is the switching frequency. The derived RFET key parameters, including the high and symmetric pn on-currents and on–off ratios, facilitate the reduction of time delay in the device due to their inverse proportionality. The stable low off-currents (*I*_OFF_) in the devices contribute to minimizing static power dissipation, particularly during the standby modes. Additional strategies to reduce delay and power consumption include optimizing the threshold voltage and lowering the supply voltage (*V*_DD_) which exhibits a quadratic dependence, enabling significant improvements in both power and delay.^73^ The nanowire channel design can also be improved by having smoother nanowires with high-quality dielectric layers and reduced contact resistance to further enhance the drive current for more energy-efficient and faster circuit performance. Similarly, diminishing the load capacitance by reducing parasitic capacitances would also enable faster switching and low-power dissipation.

In the course of this work, the reproducibility, retention, and stability of the RFET devices are analyzed across back-gate, top-gate, and two-top-gate configurations. For Si-SiO_2_-based devices, a reproducibility rate of 88.3% is achieved in the back-gated configuration, with 53 out of 60 devices showing consistent and reliable performance. Among these, 4 devices are selected for single top-gate fabrication, including 3 single-nanowire devices and one nanowire array device, all of which exhibit reproducible behavior with performances similar to those reported in the study. In the two-top-gate configuration, 5 devices are tested, showing reliable n-type modulation but limited p-type modulation (as explained earlier). To address this, modifications using hBN-SiO_2_ are implemented to improve the p-type characteristics. Retention and stability are evaluated across all configurations, with devices maintaining consistent electrical parameters, such as *I*_ON_ and on–off ratios, over multiple measurements and various time intervals. The use of hBN also protects the nanowire from environmental damage and exposure retaining its property. This is also seen in our previous work.^16^ For Si-SiO_2_–Al_2_O_3_-based devices, the reproducibility and stability are equally impressive. Of the 11 devices tested in the back-gate configuration, all exhibit consistent performance, and 3 are selected for single top-gate fabrication. These devices demonstrate good retention and stability, with no significant drift in *I*_ON_, *I*_OFF_, or on–off ratios over repeated measurements at different times. Collectively, these results highlight the robustness of the fabrication process and the reliable performance of the devices, emphasizing their suitability for practical applications.

## Experimental Section

1 × 1 cm^2^ silicon-on-insulator (SOI) substrates are used for fabricating the RFET devices. The active or top device layer of the SOI is a 20 nm intrinsic silicon (Si) layer. The nanowires are fabricated using a top-down approach due to the significance of large-scale integration, described in detail in our previous works.^16,17,74^ The process involves cleaning the substrates in Piranha solution, acetone, and isopropanol, followed by spin coating a 40 nm thick 2% hydrogen silsesquioxane (HSQ) resist, EBL exposure, and resist development.^75^ This creates the nanowire resist patterns on the top active layer. Anisotropic pattern transfer to the device layer is carried out by inductively coupled plasma reactive ion etching (ICP RIE). The residual HSQ is removed using 1% hydrofluoric acid (HF). Once the nanowires are patterned, the sample is immediately transferred to a rapid thermal oxidation (RTO) chamber for the oxidation cycles. The cycle involves a slow oxidation process of the nanowires at 875 °C for 10 min, followed by a nitrogen purge step at 875 °C for 5 min and a forming gas (9:1 mixture of nitrogen and hydrogen) annealing at 450 °C for 5 min. The gas annealing passivates the dangling bonds of the nanowire oxide by diffusing hydrogen into them. This cycle produces ca. 6–7 nm of high-quality SiO_2_ around the nanowires. For thinner oxide layers, the oxidation time is reduced. For depositing Al_2_O_3_, atomic layer deposition (ALD) is carried out with a typical cycle consisting of a 20 ms trimethylaluminum (TMA) pulse step followed by a 5 s nitrogen purge, then a 20 ms water pulse, and a final nitrogen purge step of 5 s. This ALD technique is implemented at a temperature of 300 °C, resulting in an Al_2_O_3_ film growth of 0.8 Å per cycle. To achieve an all-around deposition of ca. 6–7 nm of an Al_2_O_3_ film, a total of 75 cycles are performed. Following this, source and drain Ni contacts are fabricated through EBL patterning, HF dip to remove the native SiO_2_, UHV e-beam evaporation of Ni, and a lift-off process. Flash lamp annealing (FLA) is applied after the Ni deposition to achieve controlled Ni silicidation in the nanowire, creating atomically sharp Schottky junctions.^21^ The FLA parameters are optimized with an energy density of 89 J cm^–2^ for a pulse duration of 6 ms in a continuous nitrogen gas atmosphere. Top gates are also carefully aligned on top of the Schottky junctions by using EBL, metal deposition, and lift-off techniques. Ti and Al are used as the top gate metals. The schematic representation of the RFET fabrication process flow is illustrated in the Supporting Information in Figure S9.

## Summary

In the scope of this work, fabrication and electrical characterization of different types of scalable RFET devices are shown based on various gate dielectric materials. These devices are fabricated using an advanced millisecond-range FLA-based silicidation process that provides controlled silicidation. SiO_2_, Al_2_O_3_, and hBN are employed as gate dielectric materials. Single or double top-gate architectures are used for the characterization of the devices to exercise polarity control. SiO_2_-based devices with a single top gate demonstrated the best ambipolar results with an excellent pn on-current symmetry of 1.03 and the *I*_ON_/*I*_OFF_ ratio up to 10^8^. Al_2_O_3_-based devices also showed the lowest subthreshold swing value for the hole conduction due to their high-k properties. The unipolar nature of the RFET device is illustrated with the introduction of hBN as a passivating and encapsulating layer along with SiO_2_ to tackle issues of energy barrier induction in the nanowire channel. With further optimization of improved device design, channel materials, dielectric layers, and top gate architectures, the next generation of reconfigurable devices can be introduced. To conclude, it is seen that reconfigurable electronics have the capability to strengthen classical electronics in the future in terms of device functionality and scaling. High-performing and low-power-consuming devices can ultimately be implemented in system-level integration to open new roads in the future of integrated circuitry.

## Supporting Information Available

Unpassivated nanowire array-based device, silicidation in nanowires with SiO_2_ shell, SiO_2_ passivated nanowire array-based device, energy activation method for Schottky barrier height extraction, silicidation in nanowires with Al_2_O_3_ shell, a back-gated device with Al_2_O_3_ passivation, top-gated transfer and output characterization with varying voltages for single and nanowire array-based devices with SiO_2_ passivation and single-nanowire-based devices with Al_2_O_3_ passivation, device fabrication flowchart, and device parameter extraction (PDF)
