# Supplementary material for: High-Performance Silicon Nanowire Reconfigurable Field Effect Transistors Using Flash Lamp Annealing
Source: ACS Appl Electron Mater. 2025 Mar 6;7(6):2284–97. doi: 10.1021/acsaelm.4c01896 (PMC11948323; doi:10.1021/acsaelm.4c01896)
Supplement: Supplementary file 1 — el4c01896_si_001.pdf [file el4c01896_si_001.pdf]

# Supporting Information
# High-Performance Silicon Nanowire Reconfigurable Field Effect Transistors Using Flash Lamp Annealing

Sayantan Ghosh,[*,†,‡] Muhammad Bilal Khan,[†] Slawomir Prucnal,[†] René Hübner,[†] Phanish Chava,[†,‡] Tom Mauersberger,[¶,‡] Thomas Mikolajick,[¶,‡,§] Artur Erbe,[†,‡,§] and Yordan M. Georgiev[*,†,‖]

†*Institute of Ion Beam Physics and Materials Research, Helmholtz-Zentrum Dresden-Rossendorf (HZDR), Bautzner Landstraße 400, Dresden, 01328, Germany*

‡*Technische Universität Dresden, Dresden, 01069, Germany*

¶*Namlab gGmbH, Nöthnitzer Strasse 64, Dresden, 01187, Germany*

§*Technische Universität Dresden, Center for Advancing Electronics Dresden (CfAED), Dresden, 01069, Germany*

‖*Institute of Electronics at the Bulgarian Academy of Sciences, 72, Tsarigradsko chaussee blvd., Sofia 1784, Bulgaria*

E-mail: s.ghosh@hzdr.de; y.georgiev@hzdr.de

# Unpassivated Nanowire-Array Based Device

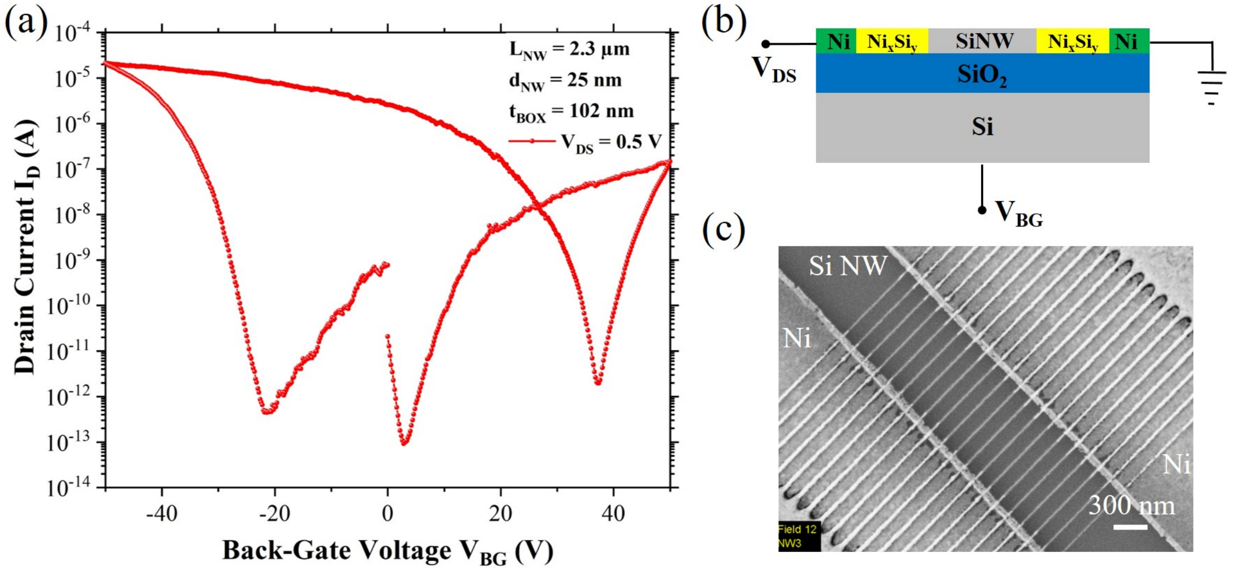


Figure S1: Unpassivated silicon nanowire-based device (a) Back gated transfer characteristics of the device having the given dimensions: effective nanowire length $L_{NW}$, diameter $d_{NW}$, and buried oxide thickness $t_{BOx}$ (b) Cross-sectional diagram of an unpassivated nanowire on SOI with the measurement scheme (c) SEM micrograph of the device.

# Silicidation in Nanowires with $SiO_2$ Shell

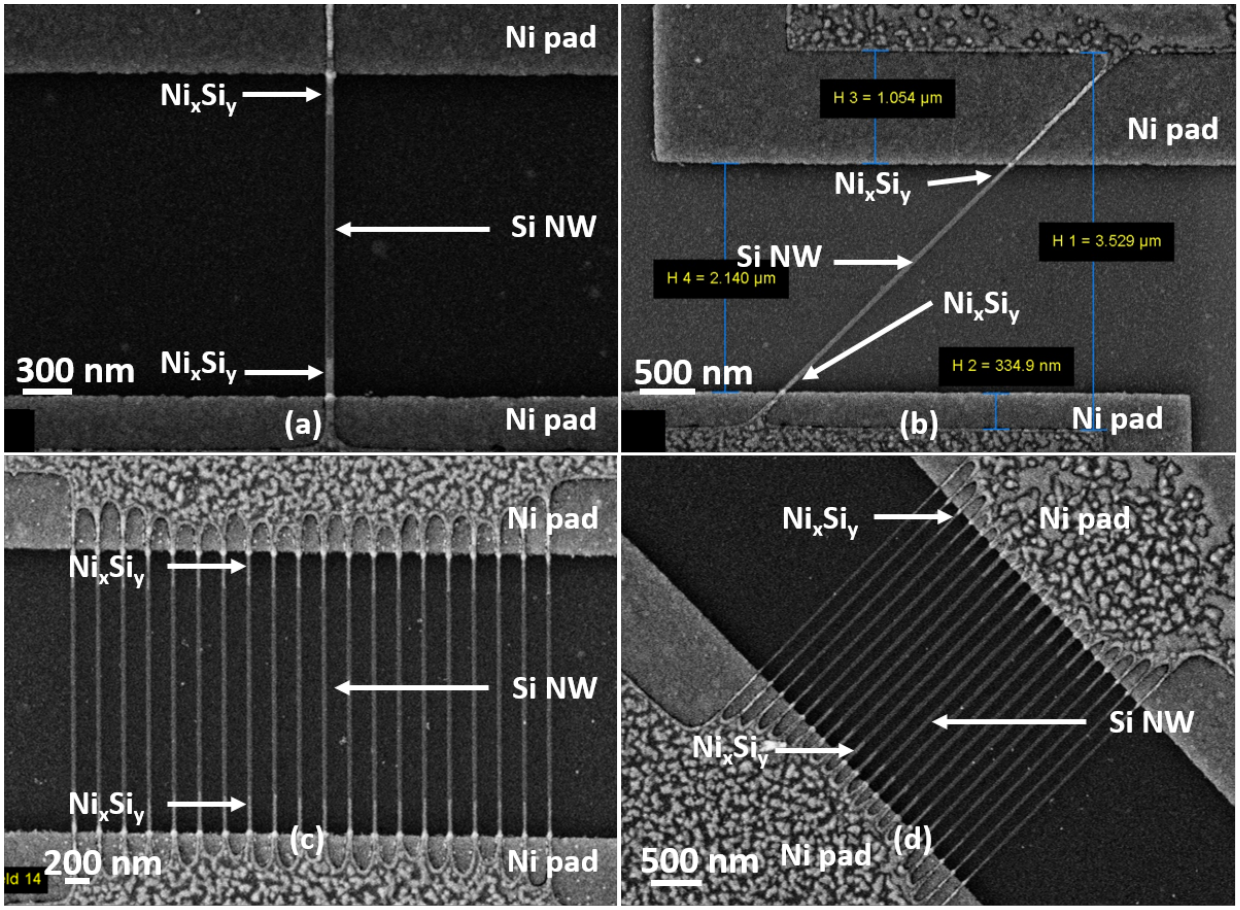


Figure S2: Top-view SEM micrographs of (a) ⟨100⟩ and (b) ⟨110⟩ oriented single nanowires, and (c) ⟨100⟩ and (d) ⟨110⟩ oriented nanowire arrays, show nanowires with a ∼ 6–7 nm $SiO_2$ shell. Silicide intrusion of up to 350 nm is observed. The annealing conditions are as follows: $N_2$ environment, 3.6 kV flash voltage, and 6 ms pulse duration.

# $SiO_2$ Passivated Nanowire-Array Based Device

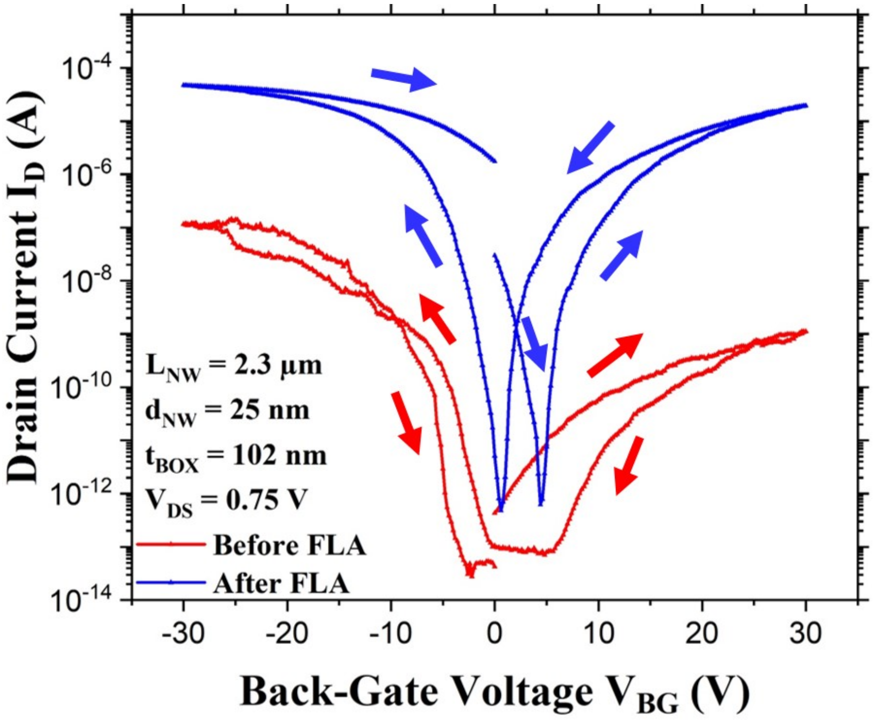


Figure S3: Back gated transfer characteristics before and after FLA for $SiO_2$ passivated nanowire array-based device at $V_{DS} = 0.75$ V

# Schottky Barrier Extraction

The Schottky barrier of the devices is calculated by the activation energy method. The drain current ($I_D$) is given by equation (1) which can be rewritten as equation (2) to draw an Arrhenius plot.[1] The linear fit of the Arrhenius plot at a certain $I_D$ has a slope of $\frac{-q(\Phi_{SB}-V_D)}{k_B T}$ and its Y intercept is $\ln(AA^{**})$. From the slope, $\Phi_B$ is calculated for various gate voltages ($V_G/V_{TG}$) (Figure **??**). $\Phi_{SB}$ corresponds to the flat band voltage ($V_{FB}$). Temperature-dependent transfer characteristics are obtained from 230 K to 340 K for calculating the Schottky barriers. Figures S3 and S4 show the transfer characteristics, Arrhenius plot, and derived Schottky barrier heights for $SiO_2$ and $Al_2O_3$ based RFET devices, respectively.

$$I_D = AA^* T^2 \exp\left(\frac{-\Phi_{SB}}{k_B T}\right) \tag{1}$$

$$\ln\left(\frac{I_D}{T^2}\right) = \ln(AA^*) - \frac{-q(\Phi_{SB} - V_D)}{k_B T} \tag{2}$$

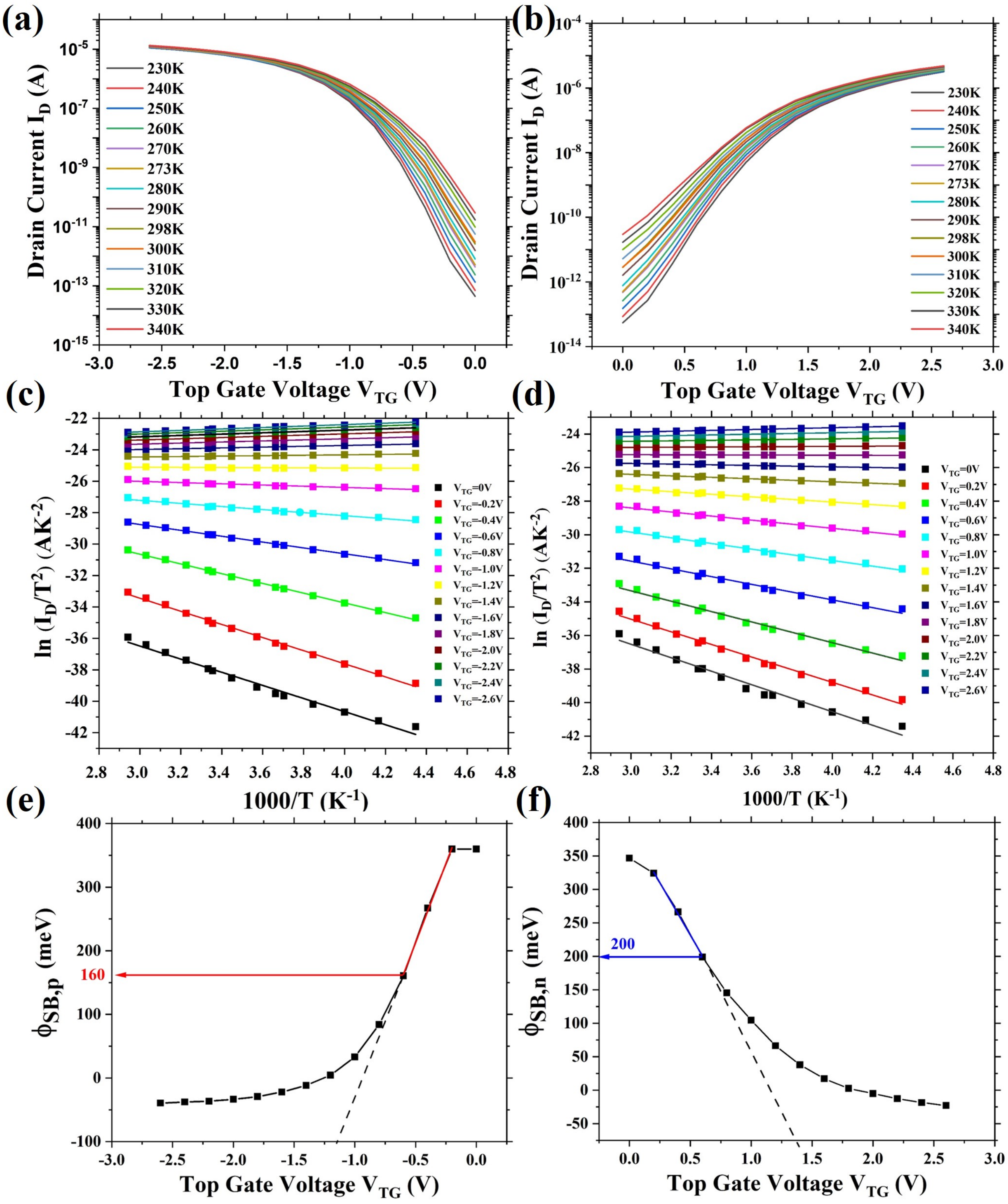


Figure S4: Temperature-dependent transfer curves (a) p-branch (b) n-branch (c, d) corresponding Arrhenius plots, and (e, f) Schottky barriers as a function of $V_{TG}$ for a single top-gated device with $SiO_2$ passivation (same device as shown in Figure 2 a,c). The extracted values of the Schottky barrier heights for the electrons and holes ($\Phi_{SB,n}$ and $\Phi_{SB,p}$) are 200 meV and 160 meV, respectively. These values are extracted from the transfer curves obtained at $V_{DS} = 1$ V.

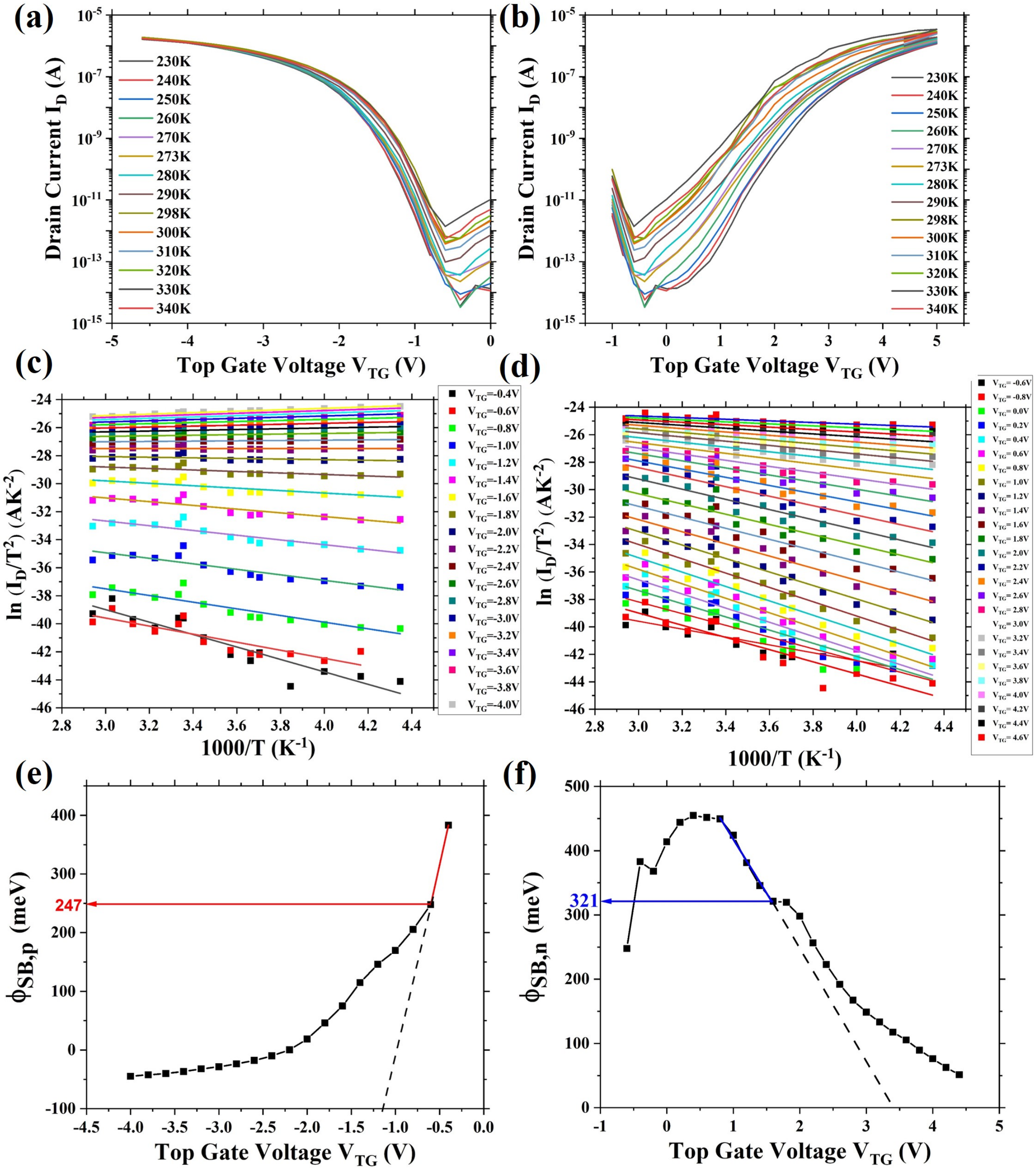


Figure S5: Temperature-dependent transfer curves (a) p-branch (b) n-branch (c, d) corresponding Arrhenius plots, and (e, f) Schottky barriers as a function of $V_{TG}$ for a single top-gated device with $Al_2O_3$ passivation (same device as shown in Figure 2 b,e). The extracted values of the Schottky barrier heights for the electrons and holes ($\Phi_{SB,n}$ and $\Phi_{SB,p}$) are 321 meV and 247 meV, respectively. These values are extracted from the transfer curves obtained at $V_{DS} = 1$ V.

## Silicidation in Nanowires with $Al_2O_3$ Shell

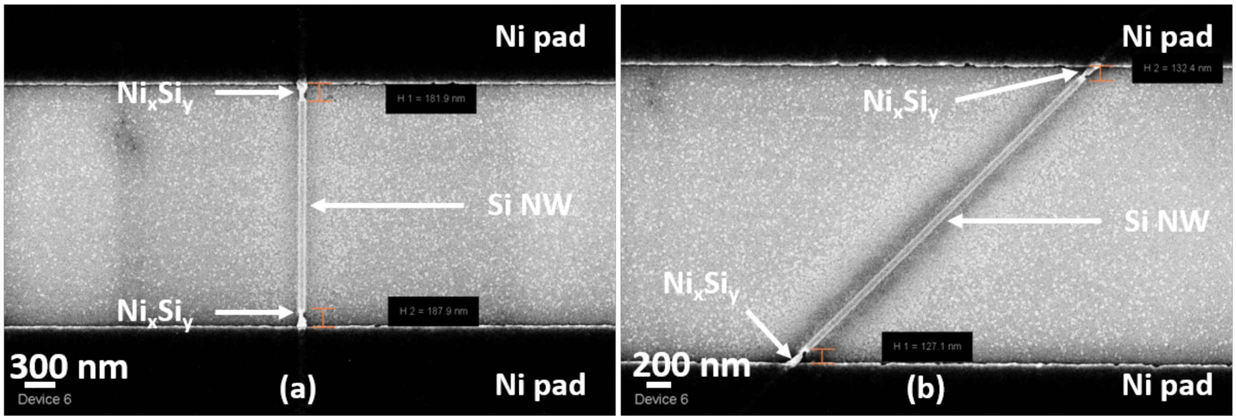


Figure S6: Top-view SEM micrographs of (a) ⟨100⟩ (b) ⟨110⟩ oriented single NWs. The nanowires are covered with a 1-2 nm $SiO_2$ and 6-7 nm $Al_2O_3$ shell. Dark notches in the silicide segments of nanowires appear to be voids. The annealing parameters are: $N_2$ environment, 3:6 kV flash voltage, and 6 ms pulse duration.

## Back-gated device with $Al_2O_3$ passivation

In this section, the back-gated device characteristics with $Al_2O_3$ passivation are discussed. Figure S7 shows the back-gated transfer characteristics, a cross-sectional layout, and a top-view SEM image of the measured single nanowire-based device.

As evident in Figure S7 (a), a typical ambipolar type of characteristics is seen. In this case, $V_{BG}$ is also swept by a butterfly sweep from -30 to 30 V, and $V_{DS}$ is kept constant at 0.75 V. Compared to the back-gated result of the unpassivated device (shown in Figure S3 (a)), this device also shows an improvement in terms of the transfer characteristics shift and the hysteresis. It is seen that the shift in the transfer curve is minimum ($I_{OFF}$ minimum centered at $V_{BG}$ of approximately 3 V) with both p- and n-type hysteresis reduced. The main reason for this is the presence of the thermally grown $SiO_2$ layer around the nanowire, which minimizes the interface-trapped charges and the other hydroxyl-charged sites. With the implementation of the dielectric stack shell around the nanowire, the symmetry of the n- and p-type branches is also enhanced. However, it can be seen that the p-type branch

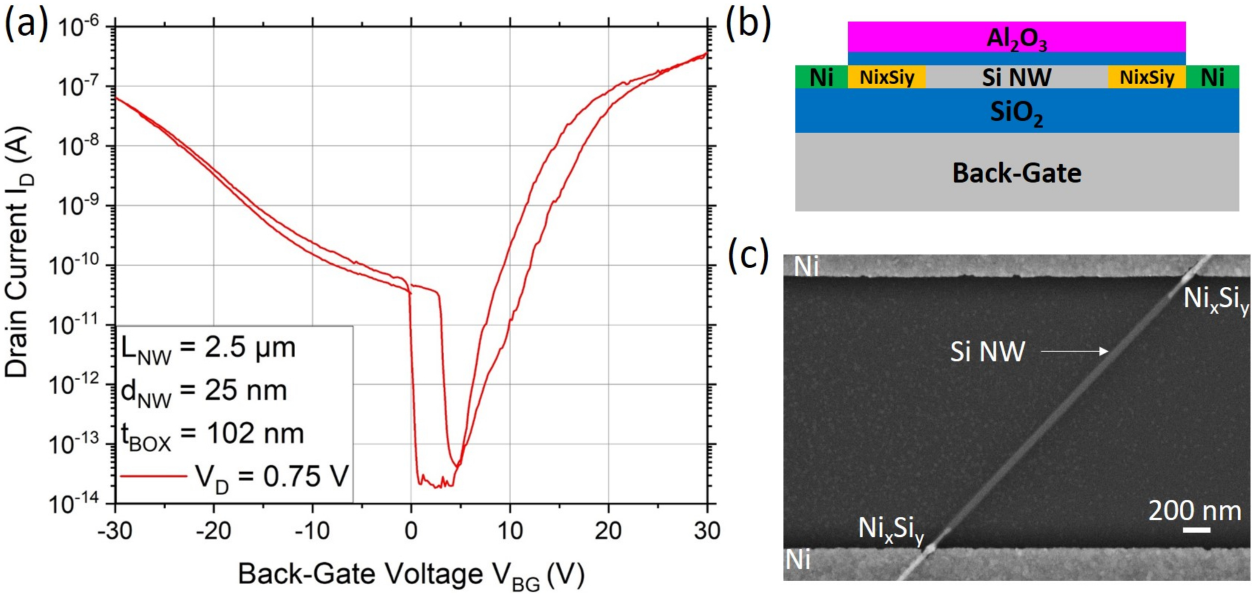


Figure S7: $Al_2O_3$ passivated single nanowire-based device (a) Back-gated transfer characteristics, (b) Cross-sectional layout, and (c) SEM micrograph of the single nanowire-based device. Lengths and widths of the nanowire are 2 $\mu$m and 25 nm, respectively.

shows a steeper curve till the origin before the slope decreases. The reason for this can be the non-uniformity of Ni silicidation of the nanowire. Since the FLA process for Ni silicidation is not optimized for the nanowires with the $Al_2O_3$ shell, further investigations are required to achieve a uniform Ni silicide progression inside the nanowires. Moreover, since the electrical measurements are carried out through the back gate, it can be presumed that the low capacitive coupling effect on the non-uniform Schottky junctions through a much thicker buried oxide degrades the subthreshold slope of the device.

Next for constant $V_{DS}$ of 0.75 V, the device parameters are extracted with the on-currents of $3.53 \times 10^{-8}$ A and $3.7 \times 10^{-7}$ A for the p- and the n-type branch, respectively. With off-currents of $3.4 \times 10^{-14}$ A for the p-type and $2.4 \times 10^{-14}$ A for the n-type, the $I_{ON}/I_{OFF}$ ratio is $\sim 10^6$ for the p-type and $\sim 10^7$ for the n-type. The subthreshold swing for the p-branch is 260 mV/dec with a threshold voltage of -17 V. For the n-branch, the subthreshold swing is 2.08 V/dec with a threshold voltage of 18 V. Finally, the recorded electron and hole mobility values for the back-gated device are 21 cm$^2$/Vs and 4.8 cm$^2$/Vs, respectively. The pn on-current symmetry of 10.48 is attained at $V_{DS}$ = 0.75 V for the device. For better

subthreshold swings and a higher capacitive coupling effect, further analysis is done with a single top gate.

## Transfer and Output characteristics of single top gated devices

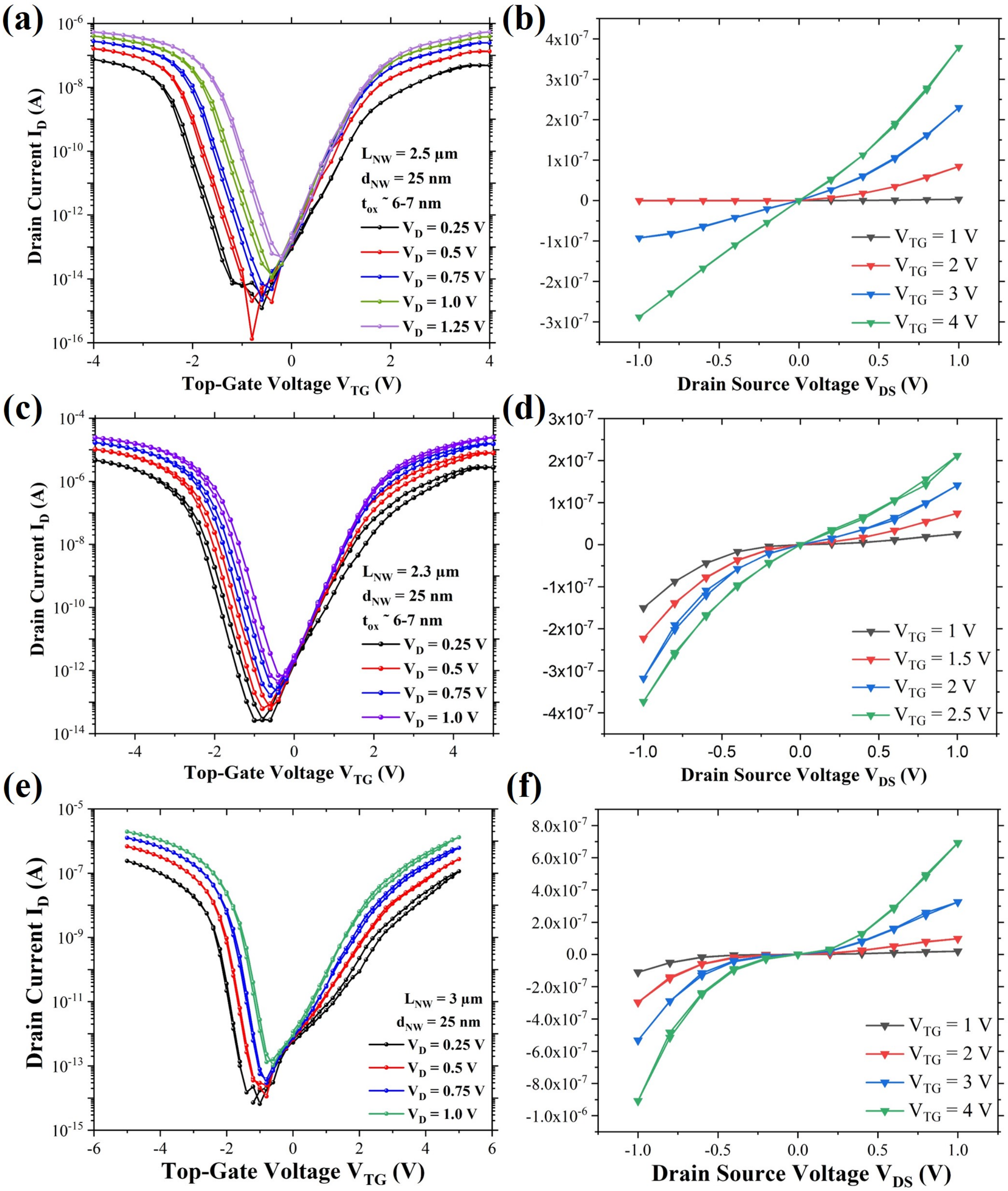


Figure S8: The top gated transfer characteristics ($I_D$-$V_{TG}$) with varying $V_{DS}$ for (a) the single nanowire-based device, (c) the nanowire array-based device consisting of 20 nanowires with $SiO_2$ passivation and (e) the single nanowire-based device with $Al_2O_3$ passivation. The output characteristics ($I_D$-$V_{DS}$) for varying $V_{TG}$ are shown for (b) the single nanowire-based device, (d) the nanowire array-based device with $SiO_2$ passivation and (f) the single nanowire-based device with $Al_2O_3$ passivation.

## Device Fabrication Flowchart

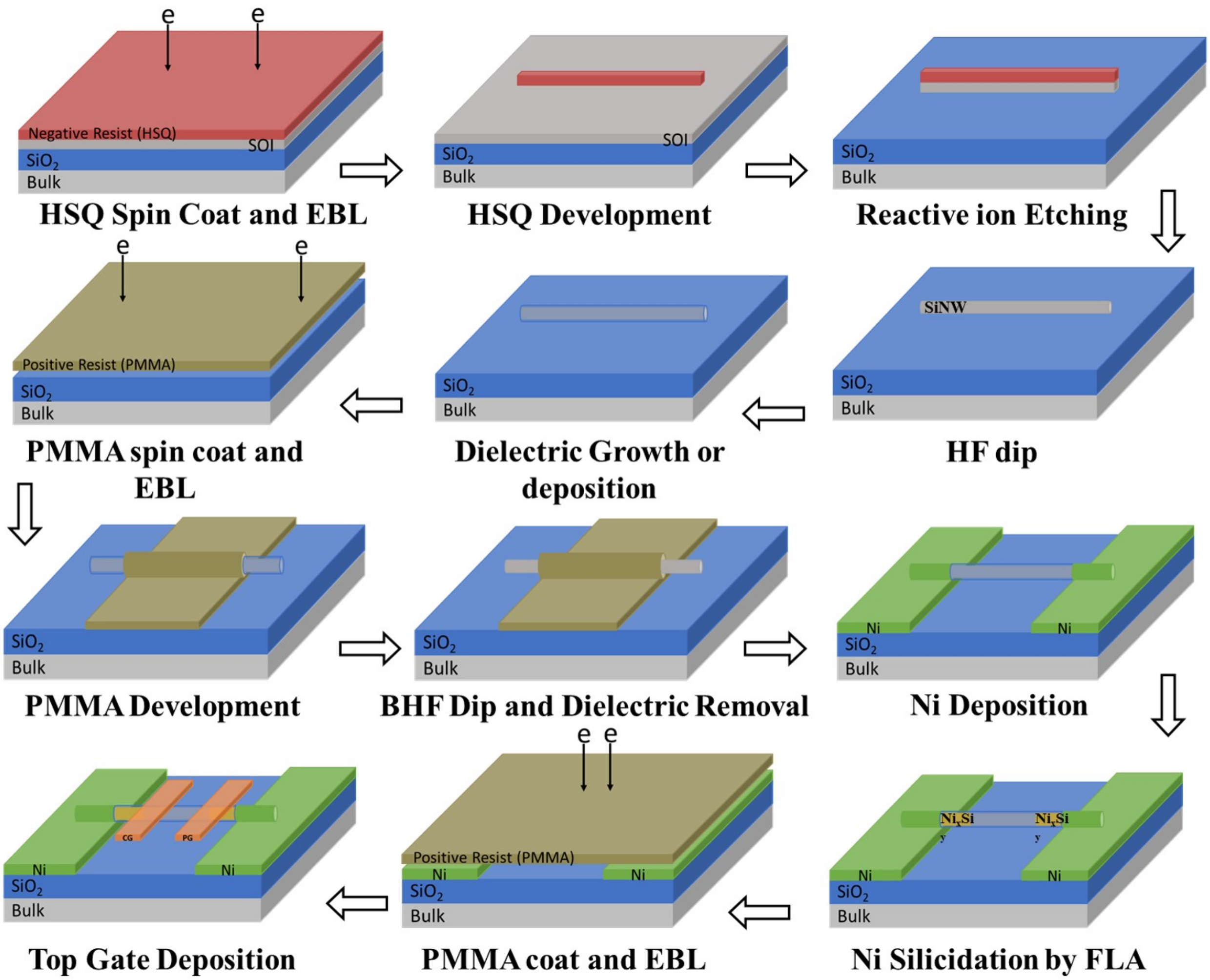


Figure S9: Schematic of the RFET fabrication process flow

# Device Parameter Extraction

## 1. On and Off-Currents

In the transfer characteristics, the on-currents ($I_{ON}$) is defined as the saturation current in the active mode of operation. The on-current for either the p-type or the n-type conductance is determined in this work from the peak current at the maximum top gate voltage ($V_{TG}$) point within the measurement range. For ideal cases, the $I_{ON}$ in the transfer characteristics is defined as the current in the active mode under the condition where the gate voltage ($V_{GS}$) and the drain-source voltage ($V_{DS}$) are similar to the supply voltage ($V_{DD}$) ($V_{GS}$=$V_{DS}$=$V_{DD}$), as this represents the relevant operating point for digital circuits. The off-current ($I_{OFF}$) is the point where the device switches off. However, there is a presence of leakage current between the source and drain terminals when the gate voltage is below the threshold voltage (in the subthreshold regime). In the case of the devices measured, there is a shift in the transfer characteristics. Hence, for the calculation of the on and the off-currents, this shift is considered. Depending on the voltage side the shift is in (either positive or negative $V_{TG}$), the peak on-current for that specific branch is calculated at the maximum voltage that can be reached. For the other branch, the voltage shift value is subtracted from its maximum voltage (opposite polarity) to determine its peak on-current. An example of the on and off-current calculation is shown with the graph S10.

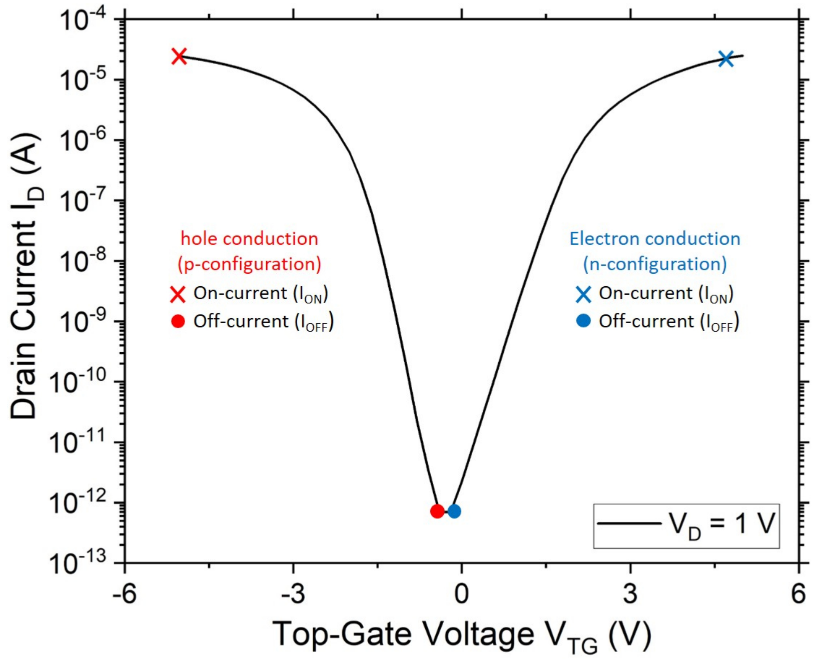


Figure S10: Calculation of on and off-current from the transfer characteristics

As seen from the graph S10, there is a shift in the transfer characteristics to a negative $V_{TG}$ (minima at 0.3 V). Therefore, the p-type on-current is calculated first at the maximum $V_{TG}$ of -5 V. $I_{ON}$ and $I_{OFF}$ of the p-branch are $2.42 \times 10^{-5}$ A and $6.97 \times 10^{-13}$ A, respectively. Now for the n-type, the shift of 0.3 V is subtracted from the maximum positive voltage ($V_{TG} = 5$ V) to determine its on-current. Hence, at $V_{TG} = 4.7$ V, the n-type on-current is calculated. For the n-branch, the $I_{ON}$ and $I_{OFF}$ are $2.33 \times 10^{-5}$ A and $6.96 \times 10^{-13}$ A, respectively. This technique of current calculation gives a fair pn on-current symmetry for equal voltage sweeps on either side. The pn on-current symmetry denotes the ratio of the maximum saturated on-current (conduction) between the p-type and n-type operations of the device. It is quantified by dividing the maximum on-current of the p-conduction by that of the n-conduction.

The pn on-current symmetry is given by the formula:

$$\text{pn on-current symmetry} = \frac{I_{\text{on, p-type}}}{I_{\text{on, n-type}}}$$

Where: $I_{\text{on, p-type}} = 2.4 \times 10^{-5}$ A, $I_{\text{on, n-type}} = 2.3 \times 10^{-5}$ A

Thus, the pn on-current symmetry is:

$$\text{pn on-current symmetry} = \frac{2.4 \times 10^{-5}}{2.3 \times 10^{-5}} \approx 1.03$$

## 2. Effective Oxide Thickness (EOT) and Oxide Capacitance

Different materials are explored and used in this work as the gate-dielectric. The dielectric constants for these materials are shown in the table S1.

Table S1: Dielectric constants of materials used in the fabrication process.

| Material | Dielectric Constant |
|---|---|
| $SiO_2$ | 3.9 |
| $Al_2O_3$ | 9.0 |
| hBN | 3.0 |

The oxide capacitance is calculated from the following equation:

$$C_{\mathrm{o}_x} = \frac{\epsilon_o \kappa A}{t_{\mathrm{o}_x}} \tag{3}$$

where, $C_{\mathrm{o}_x}$ is the oxide capacitance, $\epsilon_o$ represents the relative permittivity of free space, $\kappa$ is the dielectric constant, $A$ is per unit area and $t_{\mathrm{o}_x}$ represents the thickness of the oxide or dielectric. The equivalent oxide thickness (EOT) is calculated using the equation:

$$\mathrm{EOT} = \frac{\kappa_{\mathrm{SiO2}}}{\kappa_{\mathrm{ox}}} t_{\mathrm{ox}} \tag{4}$$

where, $\kappa_{\mathrm{SiO2}}$ is the dielectric constant of $SiO_2$, $\kappa_{\mathrm{ox}}$ and $t_{\mathrm{ox}}$ denote the dielectric constant and the thickness of the oxide or material used, respectively.

## 3. Mobility

Carrier mobility is defined as how fast a charge carrier can drift through the semiconductor or metal under the influence of an electric field. The mobility values for the electron and

the holes are calculated considering the transconductance ($g_m$) under the effect of contact resistance. This is given from the equation:

$$\mu_{p,n} = \frac{L_{\mathrm{ch}} g_m}{W_{\mathrm{ch}} C_{\mathrm{o}_x} V_{\mathrm{DS}}} \tag{5}$$

where, $L_{\mathrm{ch}}$ is the effective length of the channel, $W_{\mathrm{ch}}$ is the width of the channel, $g_m$ denotes the transconductance, $C_{\mathrm{o}_x}$ is the oxide capacitance and $V_{\mathrm{DS}}$ represents drain source voltage.

## 4. Threshold Voltage

The threshold voltage is calculated using the popular 'Extrapolation in the Linear Region (ELR)' technique.[2] It is carried out by taking the X-intercept of the linear extrapolation of the transfer curve ($\mathrm{I_D}$-$\mathrm{V_{GS}}$) slope on the $\mathrm{V_{GS}}$ axis. The slope is extracted at the maximum transconductance ($g_m$) value. The point on the X-axis ($\mathrm{I_D} = 0$) where the slope touches denotes the threshold voltage. The value of the threshold voltage is corrected by adding the term $\mathrm{V_D}/2$ to this intercept. However, the accuracy can be affected by the degradation of mobility and parasitic resistances.

## 5. Subthreshold Swing

The subthreshold slope denotes how sharply a device can be switched on. A reciprocal assessment of the subthreshold slope gives the subthreshold swing. This is defined as the change in the gate voltage required to produce a drain current of one order of magnitude. In the subthreshold regime, the drain current is exponentially related to the gate voltage. Therefore, the subthreshold swing is calculated utilizing the exponential fit with a decay equation. The subthreshold slope is expressed by $\frac{q}{nk_BT}\ln(10)$ (taken from[3]), where $n$ is the dimensionless factor. Hence, the drain current can be expressed as:[3]

$$I_D \propto e^{\frac{qV_{\mathrm{GS}}}{nk_BT}} \tag{6}$$

Equation 6 can be written in the form of an exponential decay equation as:

$$I_D = A \cdot e^{\frac{-V_{\text{GS}}}{t}} \tag{7}$$

The fitting equation for the subthreshold swing calculation is given as:

$$y = A \cdot e^{\frac{-x}{t}} + y_0 \tag{8}$$

Here, the term $t$ denotes the slope parameter. In order to represent the subthreshold swing, equation 7 is rewritten in the context of $V_{\text{GS}}$. Therefore, equation 7 can be represented as:

$$V_{\text{GS}} = -[t \cdot \ln(10)] \cdot \log_{10}(I_D) \tag{9}$$

where the subthreshold swing can be calculated from $[t \cdot \ln(10)]$. The value of $t$ is extracted from the graph, as shown in figure S11. As seen from figure S11, the transfer characteristics

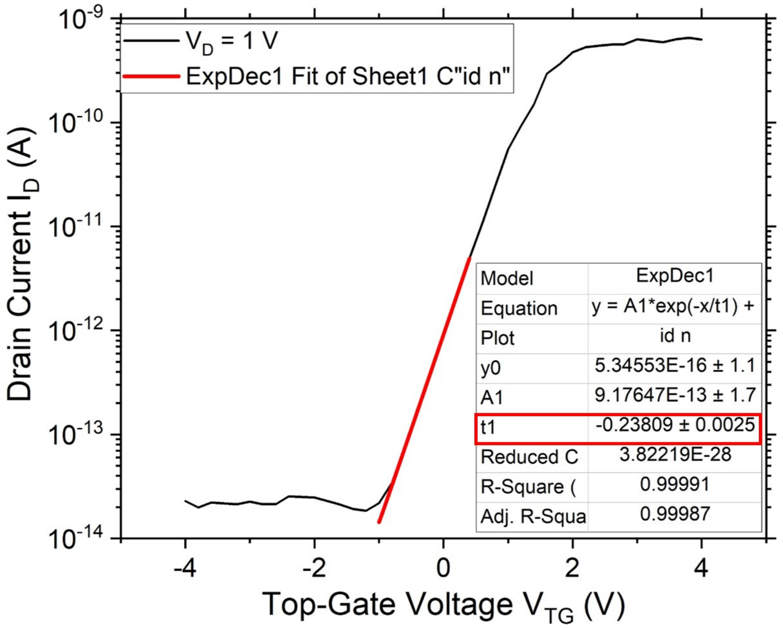


Figure S11: Calculation of subthreshold swing from the transfer characteristics

(in the subthreshold regime) are matched with the exponential fit equation to derive the value of $t$, which is -0.238. The subthreshold swing for the $n$-type curve is calculated from equation

9 by multiplying the exponential fit parameter $t$ and ln(10). Therefore, the subthreshold swing is $0.238 \times \ln(10) = 0.5481$ V/dec.

## 6. Interface Charge and Trap Density

The interface charge density ($Q_{it}$) is defined as the charge per unit area present at the Si-oxide interface due to defects and trapped carriers. The unit is expressed in C/cm$^2$. The density of traps that can capture charge carriers and affect device performance is called trap density ($D_{it}$) and is expressed in energy states (traps) per unit area per unit energy. The unit is given by cm$^2$ eV$^{-1}$. The approximate trap density is calculated based on the subthreshold swing equation by evaluating the oxide capacitance ($C_{ox}$) and the subthreshold swing (from transfer characteristics):[4]

$$D_{\mathrm{it}} = \frac{C_{\mathrm{ox}}}{q}\left(\frac{\mathrm{SS}}{\ln(10)\cdot V_T} - 1\right) \tag{10}$$

where $q$ is the elementary charge, $C_{\mathrm{ox}}$ is the oxide capacitance per unit area, $V_T$ is the thermal voltage, and SS is the subthreshold swing. The interface charge density is then calculated based on the following equation:[4]

$$Q_{\mathrm{it}} = qD_{\mathrm{it}} \tag{11}$$

# References


(1) Houssa, M.; Dimoulas, A.; Molle, A. *2D Materials for Nanoelectronics*; CRC press, 2016.

(2) Ortiz-Conde, A.; Sánchez, F. G.; Liou, J. J.; Cerdeira, A.; Estrada, M.; Yue, Y. A Review of Recent MOSFET Threshold Voltage Extraction Methods. *Microelectronics reliability* **2002**, *42*, 583–596.

(3) Sze, S. M. *Semiconductor Devices: Physics and Technology*; John wiley & sons, 2008.

(4) Sze, S. M.; Li, Y.; Ng, K. K. *Physics of Semiconductor Devices*; John wiley & sons, 2021.
